# Children Comorbidity Score, a Simple Predictor for In-hospital Mortality: A Nationwide Inpatient Database Study in Japan

**DOI:** 10.31662/jmaj.2024-0333

**Published:** 2025-04-04

**Authors:** Kayo Ikeda Kurakawa, Akira Okada, Takaaki Konishi, Nobuaki Michihata, Miho Ishimaru, Hiroki Matsui, Kiyohide Fushimi, Hideo Yasunaga, Toshimasa Yamauchi, Masaomi Nangaku, Takashi Kadowaki, Satoko Yamaguchi

**Affiliations:** 1 Department of Prevention of Diabetes and Lifestyle-Related Diseases, The University of Tokyo, Tokyo, Japan; 2 Department of Breast and Endocrine Surgery, Graduate School of Medicine, The University of Tokyo, Tokyo, Japan; 3 Department of Clinical Epidemiology and Health Economics, School of Public Health, The University of Tokyo, Tokyo, Japan; 4 Cancer Prevention Center, Chiba Cancer Center Research Institute, Chuo-ku, Japan; 5 Department of Dental Public Health, Graduate School of Medical and Dental Sciences, Institute of Science Tokyo, Tokyo, Japan; 6 Department of Health Policy and Informatics, Institute of Science Tokyo, Tokyo, Japan; 7 Department of Diabetes and Metabolism, Graduate School of Medicine, The University of Tokyo, Tokyo, Japan; 8 Department of Nephrology and Endocrinology, Graduate School of Medicine, The University of Tokyo, Tokyo, Japan; 9 Toranomon Hospital, Tokyo, Japan

**Keywords:** Children Comorbidity Score, in-hospital mortality, pediatric database study

## Abstract

**Introduction:**

Utilizing a nationwide inpatient database in Japan, we aimed to develop a novel comorbidity score for pediatric patients to predict in-hospital mortality―the Children Comorbidity Score (CCS)―based on the International Classification of Diseases, 10th Revision (ICD-10) codes.

**Methods:**

We retrospectively analyzed pediatric patients hospitalized between 2010 and 2017 using the Japanese Diagnosis Procedure Combination database. Eighty percent of the data was used as a training set, where we applied Lasso regression to a model with 56 candidate comorbidity categories to predict in-hospital mortality. We employed the 1-standard-error rule in Lasso regression to derive a parsimonious model and forced the entry of 12 categories of pediatric Complex Chronic Conditions (CCC). Thus, we developed the CCS, an integer-based comorbidity score using the selected variables with nonzero coefficients. The remaining 20% of the data was used as the test set, where we evaluated the CCS’s predictive performance using C-statistics, calibration, and decision curve analysis, comparing it with two other scores: a CCC-based score using ICD-10 codes and the Charlson Comorbidity Index (CCI).

**Results:**

Among 1,968,960 pediatric patients, we observed 6,492 (0.33%) in-hospital mortalities. The developed integer-based CCS, utilizing 10 comorbidity categories via variable selection by Lasso regression, had better discrimination ability (C-statistics, 0.720 [95% confidence intervals (CI), 0.707-0.734]) than the CCC (0.649 [0.636-0.662]) and CCI (0.544 [0.533-0.555]). The superior discrimination of the CCS was consistent across all age categories, sexes, and body mass index categories. The CCS showed good calibration, with a calibration slope of 1.027 (95% CI, 0.981-1.073). Decision curve analysis indicated that the CCS provided the highest net benefit compared to either of the reference models.

**Conclusions:**

The ICD-10-based CCS outperformed conventional comorbidity scores in predicting in-hospital mortality and would be useful in comorbidity assessment among pediatric inpatients.

## Introduction

Administrative databases are frequently utilized for retrospective cohort studies because they enable researchers to conduct large-scale studies at lower costs than those of randomized controlled trials and prospective cohort studies. Analysis of administrative databases is particularly useful for pediatric populations because conducting clinical trials or cohort studies involving children is less feasible than among adults, owing to smaller target populations and ethical concerns ^(1), (2), (3), (4)^.

Despite the high viability of incorporating administrative databases into epidemiological studies, adjusting for comorbidities and patient demographics is essential to reduce the bias inherent in such studies ^(5)^. In adult populations, several comorbidity scores, including the Charlson Comorbidity Index (CCI) ^(6), (7)^ and Elixhauser Comorbidity Index (EC) ^(8)^, have been used for risk adjustment in administrative database studies ^(5), (9), (10)^. These scores are efficacious because they are defined by the International Classification of Diseases, 10th Revision (ICD-10) codes and summarize various comorbidities into a single weighted parameter.

Comorbidity assessment tools for pediatric populations are scarce, with the pediatric complex chronic conditions (CCC) classification system being one of the limited options available ^(11), (12)^. The CCC classification system encompasses 12 disease categories and has been used for risk adjustment in several pediatric database studies ^(13), (14)^. However, disadvantages in the use of the CCC classification system include the lack of weighting of comorbid conditions and its limited applicability due to CCC being defined by the ICD-10 clinical modification (ICD-10-CM), which is used exclusively in the United States. Moreover, the CCC were originally developed as a disease complex for classifying chronic comorbidities related to pediatric mortality ^(11), (15), (16)^, rather than as a specific risk score. Therefore, when used for risk evaluation, the application of the CCC varies ^(17), (18), (19)^, with some approaches using the total number of categories, whereas others assess based on whether at least one category is applicable. Apart from CCC, which was originally developed for classifying comorbidities, there exists a risk score called the Pediatric Comorbidity Index that was developed for predicting one-year risk of hospitalization ^(20)^. However, the Pediatric Comorbidity Index is also calculated using ICD-10-CM codes, which limits its applicability outside the United States. Consequently, scoring systems based on ICD-10 codes are warranted to reflect clinically important diseases in pediatric populations, similar to the CCI or EC in adult populations.

This study aimed to develop the Children Comorbidity Score, a weighted integer risk score for predicting pediatric in-hospital mortality, utilizing a nationwide inpatient database in Japan. The Children Comorbidity Score is based on a parsimonious machine learning model employing Lasso regularization. We also evaluated the predictive ability, calibration, and clinical effectiveness of the model by comparing its performance with the corresponding ICD-10-defined CCC ^(11)^ and CCI scores.

While including factors such as admission-precipitating diagnosis, age, sex, and in-hospital procedures would likely improve the predictive ability of the model for in-hospital mortality ^(21)^, the goal of this study was not to create a highly predictive model. Instead, we focused on developing a simple score based solely on comorbidities, aiming to provide a practical tool that can be easily applied in clinical settings.

## Materials and Methods

### Data source

This study utilized data from the Diagnosis Procedure Combination database, a nationwide inpatient database in Japan. The details of this database have been previously documented ^(22), (23)^. The database contains inpatient administrative claims and discharge abstracts for more than 90% of all tertiary care emergency departments. The participating hospitals were distributed across all 47 prefectures in Japan. The database includes the following information: patient age, sex, height, body weight, six types of diagnoses based on ICD-10 codes (main diagnosis, admission-precipitating diagnosis, most resource-consuming diagnosis, second most resource-consuming diagnosis, comorbidities present on admission, and complications arising after admission), medical procedures, medications and devices used, and discharge status (discharge home, discharge to another hospital, or in-hospital mortality).

The study protocol was approved by the Institutional Review Board of the Graduate School of Medicine, The University of Tokyo (2018030NI). As the study relied on anonymized retrospective data, the requirement for informed consent from individual patients was waived.

### Study population and eligibility criteria

We included all patients aged 3-17 years who were admitted to the participating hospitals between July 2010 and March 2017. We selected the last admission for patients with more than one hospital admission during the study period. We excluded patients who were transferred to another hospital within 2 days of admission ^(9), (24)^ and patients with missing information on the admission-precipitating diagnosis or discharge outcome.

### Study variables and outcome

#### Variables other than diseases and outcomes used in this study

We collected data on age, sex, height, weight, use of ambulance on admission, admission type (scheduled or unscheduled), and all registered diagnoses. Age was categorized into three groups: 3-5, 6-12, and 13-17 years. The body mass index (BMI) was categorized using cutoffs of 18.5 kg/m^2^ and 25.0 kg/m^2^ in patients aged 17 years. For patients younger than 17 years, the BMI was categorized according to the BMI standard deviation score (BMI-SDS). As previously described ^(25)^, the BMI-SDS was calculated using BMI, sex, and age in months to assess BMI in an age- and sex-dependent manner. The patients were classified as underweight (BMI-SDS < −1.28), normal weight (BMI-SDS −1.28 to 1.279), and overweight or obese (BMI-SDS ≥ 1.28) ^(26)^. We created a “missing” category and reported the proportions of missing values for BMI, use of ambulance on admission, and admission type.

The outcome of interest was in-hospital mortality, defined as death from any cause during hospitalization.

#### Twelve primary comorbidity categories

We defined 12 primary comorbidity category names as used in the ICD-10-CM-defined CCC categories: neurological and neuromuscular, cardiovascular, respiratory, renal and urologic, gastrointestinal, hematologic and immunologic, metabolic, other congenital and genetic, malignancy, premature and neonatal, use of medical devices, and transplantation. Due to the transplantation category in the ICD-10-CM-defined CCC primarily being defined using specific ICD-10-CM codes, we categorized T86 and Z94 as the transplantation category within the primary comorbidity categories ^(27)^. We included codes for the 12 primary comorbidity categories by combining the ICD-10-defined CCC categories with the corresponding categories in the CCI and EC. The ICD-10-defined CCC categories present on admission were identified using the CCC V2 Stata Program ^(11)^ in circumstances where all diseases were exclusively documented using ICD-10, and not ICD-10-CM codes. To encompass a wider range of important comorbidities (i.e., those associated with the 12 categories and in-hospital mortality not addressed by the ICD-10-defined CCC, CCI, or EC), we introduced additional comorbidities within the 12 categories. These were selected from the 100 most frequently occurring comorbidities identified using two-digit ICD-10 codes among patients who died in the hospital. Supplementary methods showed the process of preparation of 12 primary comorbidity categories.

#### Forty-four comorbid subcategories

We established 44 comorbid subcategories by building upon the existing ICD-10-defined CCC categories, 17 CCI categories, and 31 EC categorizations. This expansion allowed us to obtain more detailed information, including disease severity and etiology. To create these 44 subdivided categories, we merged similar diseases into a single category and made clinical revisions while retaining the original classification of the ICD-10-defined CCC, CCI, and EC. Supplementary methods showed the process of preparation of the subdivided categories. Supplementary Table 1 presents the ICD-10 codes for the 44 comorbid subcategories. Since comorbidities were identified based on the presence of corresponding codes, no missing data were observed, as cases with no recorded codes were classified as having no comorbidities.

### Statistical analysis

#### Descriptive analysis

We summarized the background characteristics of the pediatric patients, stratified by in-hospital mortality status. Categorical variables were compared using the chi-square test, whereas continuous variables were compared using the *t*-test or Mann-Whitney test.

To describe the type of admission, we categorized each hospitalization into groups based on the ICD-10 code for the admission-precipitating diagnosis. We described the in-hospital mortality rate for each hospital.

#### Population splitting into model development and model validation

The patients were randomly divided into a training set (80% of the total population) and a test set (the remaining 20% of the total population). We used standardized differences to assess differences in patient characteristics between the training and the test set. An absolute standardized difference of > 0.1 indicated imbalance ^(28)^.

Using the training set, we developed a comorbidity score for predicting in-hospital mortality and validated the developed score using the test set.

#### Identifying risk factors and developing a prediction model

Among the 56 candidate variables, we applied the one-standard-error (1SE) rule of the Lasso regression to predict in-hospital mortality, as in previous studies using logistic regression with the 1SE rule ^(29), (30), (31), (32)^. We included the 12 primary comorbidity categories as mandatory features and employed 5-fold cross-validation. Lasso regression, known for its efficiency, combines variable shrinkage with enhanced interpretability ^(33)^. The 1SE rule entails selecting the maximum hyperparameter λ within a 1SE range of the difference, leading to a more parsimonious model.

Using the selected variables, we calculated an integer-based comorbidity score by multiplying the coefficients obtained from logistic regression by a scaling factor ^(34), (35)^. We named this resulting score the “Children Comorbidity Score (CCS).”

#### Model validation using C-statistics, calibration, and decision curve analysis

In the test set, we measured predictive performance using C-statistics (area under the receiver operating characteristic curve, comprising plots of sensitivity vs. 1 minus specificity to assess the discriminative ability of each marker). The C-statistics between the models were compared using the DeLong test with Bonferroni correction.

Subsequently, we evaluated the model performance using the test set in terms of calibration. Calibration was used to assess the consistency between predicted probabilities and actual diagnosis. This included assessing (i) the calibration-in-the-large (CITL) index, which describes the difference between the average predicted probabilities and the observed event frequencies, with an ideal value of zero, and (ii) the calibration slope, with an ideal value of one. The 95% confidence intervals (CIs) of the CITL and slope were calculated using the bootstrap method.

The clinical effectiveness of the models for predicting in-hospital mortality was evaluated using decision curve analysis, which considers net benefit across threshold probability ^(36)^. Decision curve analysis evaluates the trade-offs between correct mortality predictions (true positives) and unnecessary preparations (false positives). The threshold probability, shown on the x-axis, is the point where the benefit of intervention outweighs the risks. Higher thresholds reduce false positives but may miss true cases, whereas lower thresholds identify more true positives but increase false positives. The threshold probability reflects clinical preferences, where clinicians choose a lower threshold to accept more false positives to ensure intervention and a higher threshold for avoiding unnecessary treatment. The y-axis represents the net benefit, balancing correct interventions against unnecessary ones.

In pediatric mortality prediction, lower threshold probabilities are important because missing a fatal case (false negative) in children can be more harmful. At the same time, unnecessary interventions, including close observation in an intensive care unit, are generally less invasive and acceptable. Each model was compared with two extreme default scenarios: providing preventive care for all patients (i.e., treat all, which would result in preparing all patients for the risk of in-hospital mortality regardless of their risk, ensuring no high-risk cases are missed but leading to unnecessary interventions for low-risk patients) or providing preventive care for no patients (i.e., treat none, which would avoid unnecessary interventions but could fail to support any high-risk patients). This method incorporates considerations of discrimination and calibration, expanding the evaluation of models to include their potential impact on clinical decision-making.

We also performed subgroup analyses stratified by age, sex, and BMI categories and compared the C-statistics between the developed CCS and the reference models.

#### Reference models

For the reference models, we utilized the total number of ICD-10-defined CCC categories (the sum of the number of CCC categories without considering the weight of each category) or CCI. In a sensitivity analysis, we employed the model using all ICD-10-defined CCC categories, considering the weight of each category instead of their total numbers.

The significance threshold was set at p < 0.05. All statistical analyses were conducted using Stata, version 18 (Stata Corp., College Station, TX, USA).

## Results

### Preparation of the 12 primary comorbidity categories

Supplementary Table 2 presents the 100 most commonly recorded ICD-10 codes as comorbidities on admission among patients who died in the hospital. Among these, we identified four ICD-10 codes for chronic comorbidities not covered in the ICD-10-defined CCC, CCI, or EC: D64 (other anemias), K21 (gastroesophageal reflux disease), Q87 (other specified congenital malformation syndromes affecting multiple systems), and F79 (unspecified mental retardation). Therefore, we added these four codes to the 12 primary comorbidity categories. The details of the developed 12 primary comorbidity categories are presented in Supplementary Table 3.

### Descriptive analysis

**Figure 1.**
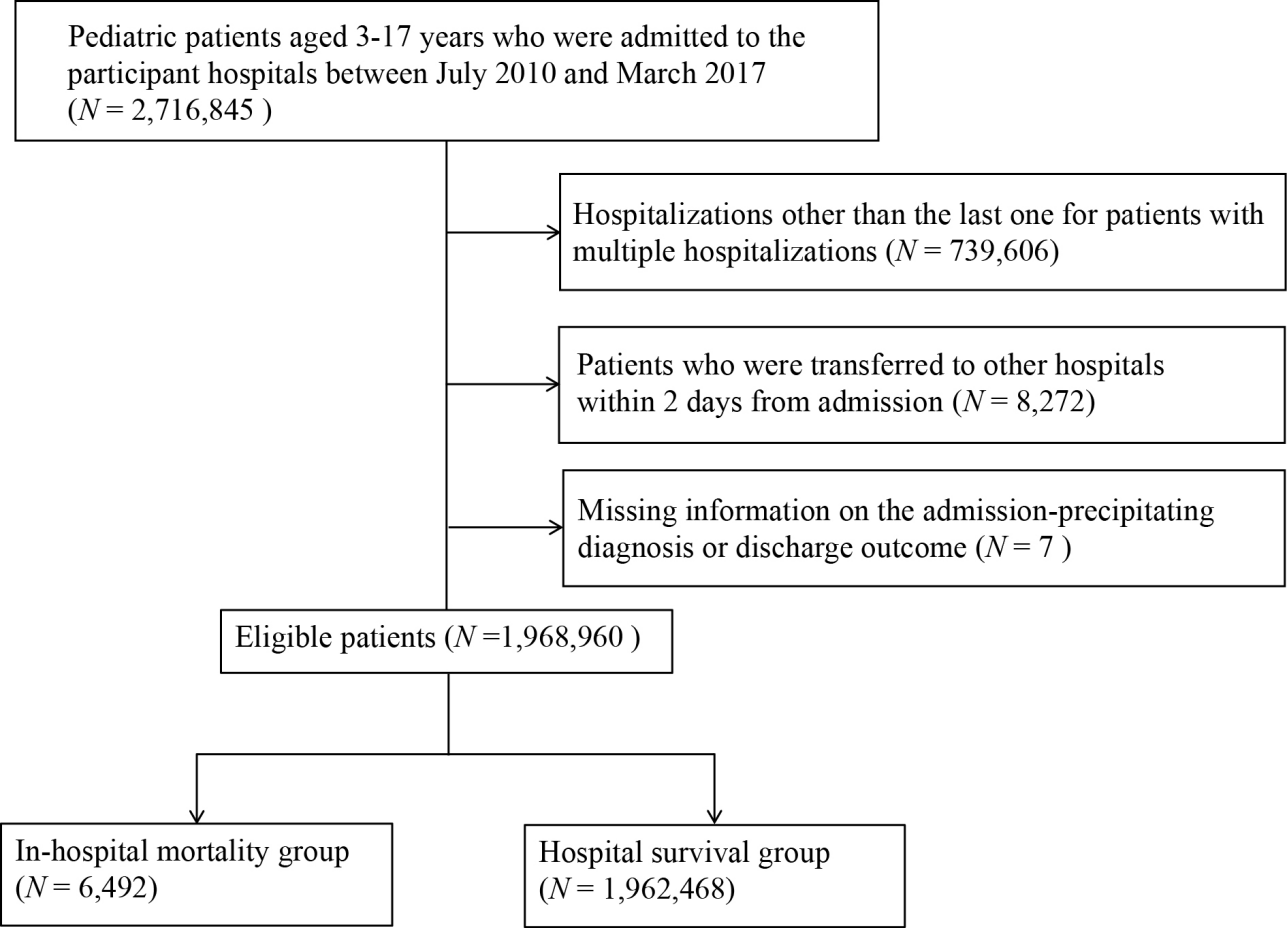
Flow chart showing the patient selection process.

During the study period, 2,716,845 pediatric patients were identified from the database. Among them, we excluded 747,885 patients according to the exclusion criteria (Figure 1). The remaining 1,968,960 patients from 1,625 hospitals were included in this study. We identified 6,492 in-hospital mortalities (0.33%) in the database.

Table 1 presents the clinical characteristics of the patients stratified by in-hospital mortality status. Patients who died during hospitalization were more likely to be male (59.7% vs. 57.5%, p < 0.001) and older (11 vs. 8 years, p < 0.001). Regarding BMI, the proportions of patients who were underweight (22.9% vs. 14.4%, p < 0.001) and those with missing values (35.8% vs. 9.0%, p < 0.001) were higher among patients who died during hospitalization. The proportions of patients who used ambulance services (58.6% vs. 9.8%, p < 0.001) and those who had unscheduled admissions (80.9% vs. 53.5%, p < 0.001) were also higher among patients who died during hospitalization. Regarding the 12 primary comorbidity categories, the proportion of patients with comorbidities such as neurological and neuromuscular disorders, cardiovascular diseases, renal and urologic conditions, gastrointestinal diseases, hematologic and immunologic disorders, metabolic disorders, other congenital and genetic defects, malignancies, premature and neonatal disorders, use of medical devices, and transplantation was higher among patients who died during hospitalization. In contrast, the proportion of patients with respiratory diseases was lower among patients who died during hospitalization (4.7% vs. 7.4%, p < 0.001). The CCI (0.52 vs. 0.11, p < 0.001) and total number of CCC (0.45 vs. 0.05, p < 0.001) were higher among patients who died during hospitalization.

**Table 1.** Clinical Characteristics of Hospitalized Pediatric Patients, Stratified by In-Hospital Mortality Status.

	Hospital survival group (N=1,962,468)		In-hospital mortality group (N=6,492)		*P*-value
Sex (male)	1,128,908	57.5%	3,875	59.7%	<0.001
Age (years, median, IQR)	8	5-13	11	6-15	<0.001
Age (years)					
3-5	639,177	32.6%	1,402	21.6%	<0.001
6-11	685,880	34.9%	1,938	29.9%	
12-17	637,411	32.5%	3,152	48.6%	
Body mass index					
Underweight	282,348	14.4%	1,487	22.9%	<0.001
Normal	1,328,987	67.7%	2,120	32.7%	
Overweight and obesity	174,426	8.9%	558	8.6%	
Missing	176,707	9.0%	2,327	35.8%	
Use of ambulance on admission					
No	1,768,577	90.1%	2,667	41.1%	<0.001
Yes	193,229	9.8%	3,804	58.6%	
Missing	662	0.0%	21	0.3%	
Unscheduled admission					
No	911,515	46.4%	1,213	18.7%	<0.001
Yes	1,050,277	53.5%	5,254	80.9%	
Missing	676	0.0%	25	0.4%	
Comorbidities					
Neurological and neuromuscular	62,258	3.2%	1,393	21.5%	<0.001
Cardiovascular	37,840	1.9%	863	13.3%	<0.001
Respiratory	145,696	7.4%	304	4.7%	<0.001
Renal and urologic	5,543	0.3%	84	1.3%	<0.001
Gastrointestinal	37,409	1.9%	358	5.5%	<0.001
Hematologic and immunologic	25,770	1.3%	722	11.1%	<0.001
Metabolic	145,056	7.4%	634	9.8%	<0.001
Other congenital and genetic	14,947	0.8%	209	3.2%	<0.001
Malignancy	10,012	0.5%	494	7.6%	<0.001
Premature and neonatal	628	0.0%	17	0.3%	<0.001
Use of medical devices	3,878	0.2%	144	2.2%	<0.001
Transplantation	2,788	0.1%	167	2.6%	<0.001
Charlson Comorbidity Index (mean, SD)	0.11	0.39	0.52	1.42	<0.001
Total number of CCC (mean, SD)	0.05	0.26	0.45	0.72	<0.001

ICD-10, International Classification of Diseases, 10th revision; CCC, complex chronic conditions; IQR, interquartile range; SD, standard deviation.

Supplementary Table 4 presents the categories of the admission-precipitating diagnoses of the patients in this study. Among these diagnostic categories, the most common admission-precipitating diagnoses included respiratory diseases (ICD-10 code J, 24.6%), injury/poisoning (ICD-10 codes S, T, 17.4%), and digestive diseases (ICD-10 code K, 10.1%). Supplementary Figure 1 shows the average mortality rate for each hospital. Out of 1,625 hospitals, 956 (59%) reported an average in-hospital mortality rate of zero, while fewer than 5% had an average in-hospital mortality rate exceeding 0.01.

The difference in patients’ characteristics between the training and test sets was not significant (Supplementary Table 5).

### Development of an integer-based pediatric comorbidity risk score for in-hospital mortality

**Figure 2.**
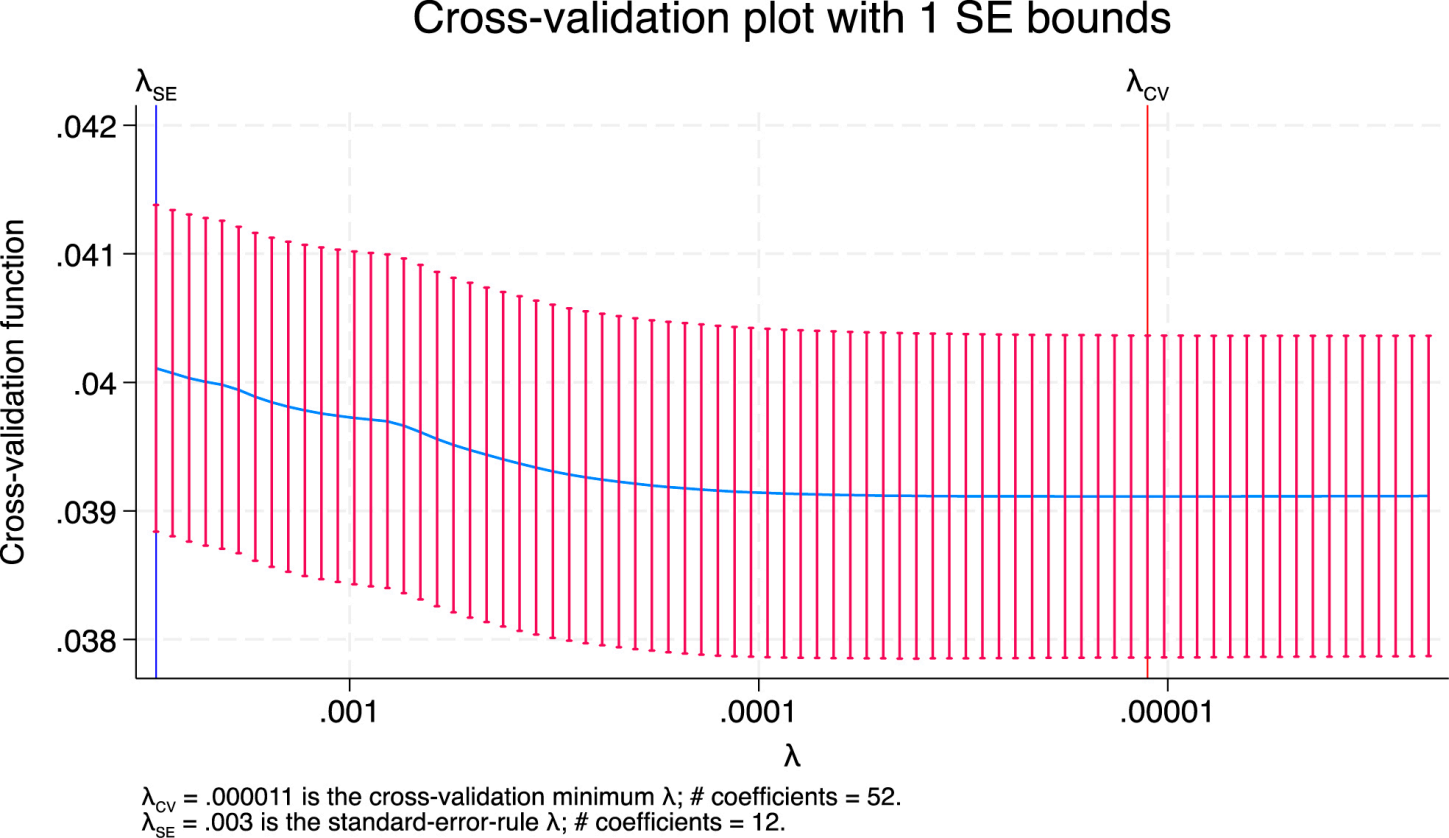
Cross-validation plot of mean squared error corresponding to smoothing parameter λ with one standard error. Cross-validation plot of mean squared error corresponding to smoothing parameter λ with standard errors. λSE, the largest λ among λ for which the cross-validation function is within one standard error of the minimum of the cross-validation function (λ = 0.003); λCV, λ where the cross-validation function is minimum (λ = 0.000011)

The Lasso regression model, with a forced entry of the 12 primary comorbidity categories, did not include any other comorbidity category as a predictor of in-hospital mortality (Figure 2). To create an integer score, we multiplied the regression coefficients by a scaling factor and rounded them to the nearest integer. We used a scaling factor of four. The weights assigned to the 12 primary comorbidity categories are detailed in Table 2.

**Table 2.** Regression Coefficients and Assigned Weights for the 12 Primary Comorbidity Categories for Predicting In-Hospital Mortality.

Category	Regression coefficients	95% CI			*p*-value	Assigned weight
Neurological and neuromuscular	1.954	1.884	-	2.024	< 0.001	8
Cardiovascular	1.547	1.460	-	1.634	< 0.001	6
Respiratory	-0.628	-0.760	-	-0.497	< 0.001	-3
Renal and urologic	0.007	-0.283	-	0.297	0.960	0
Gastrointestinal	0.237	0.104	-	0.370	< 0.001	1
Hematologic and immunologic	1.741	1.646	-	1.836	< 0.001	7
Metabolic	0.091	-0.005	-	0.186	0.063	0
Other congenital and genetic	0.862	0.700	-	1.023	< 0.001	3
Malignancy	2.407	2.296	-	2.518	< 0.001	10
Premature and neonatal	1.168	0.655	-	1.681	< 0.001	5
Use of medical devices	0.997	0.775	-	1.219	< 0.001	4
Transplantation	1.516	1.298	-	1.735	< 0.001	6

CI, confidence interval.

Among these categories, the renal and urologic, and metabolic categories were assigned weights of zero. Consequently, the CCS was computed as the sum of the assigned weights for 10 categories: neurological and neuromuscular, cardiovascular, respiratory, gastrointestinal, hematologic and immunologic, other congenital and genetic, malignancy, premature and neonatal, use of medical devices, and transplantation. To prevent negative values in the overall CCS, we added three because the assigned weight for the respiratory comorbidity category was negative.

### Model validation using C-statistics, calibration, and decision curve analysis

**Figure 3.**
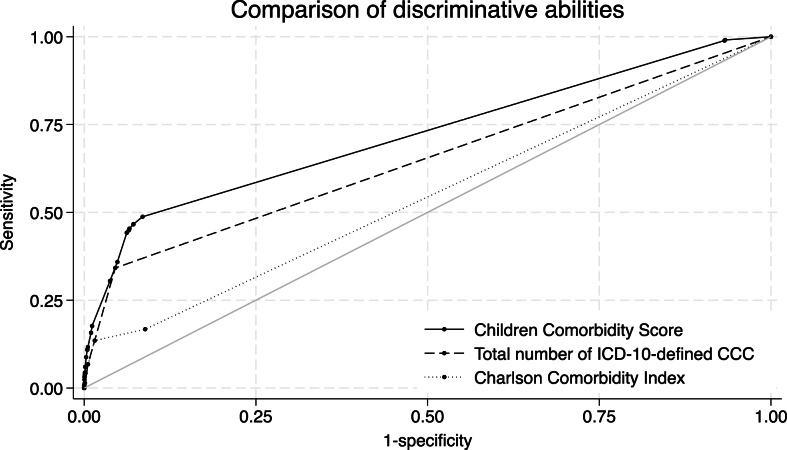
Predictive performance of the developed model (Children Comorbidity Score) and reference models for in-hospital mortality. Predictive performance of the developed Children Comorbidity Score, total number of ICD-10-defined CCC, and Charlson Comorbidity Index. The receiver operating curves for predicting in-hospital mortality in the validation cohort are shown. The corresponding area under the curve values for each model are listed in Table 3.

The receiver operating curves of the developed and reference models are shown in Figure 3. As shown in Table 3, the CCS showed better discrimination ability (C-statistic, 0.720 [95% CI, 0.707-0.734]) than the reference models employing the total number of CCC (0.649 [0.636-0.662], p < 0.001) or CCI (0.544 [0.533-0.555], p < 0.001). The C-statistics of the logistic regression model using all 56 comorbidity categories was 0.733 (95% CI, 0.719-0.747).

**Table 3.** Discrimination Ability of the Children Comorbidity Score, Total Number of ICD-10-Defined CCC, and Charlson Comorbidity Index.

		C-statistics (95% CI)	*p*-value
Developed model	Children Comorbidity Score	0.720 (0.707-0.734)	Ref
Reference models	Total number of ICD-10-defined CCC	0.649 (0.636-0.662)	< 0.001
	Charlson Comorbidity Index	0.544 (0.533-0.555)	< 0.001

ICD-10, International Classification of Diseases, 10th revision; CCC, complex chronic conditions; CI, confidence interval.

For calibration, two markers in the developed integer-based CCS yielded comparable results to those obtained with the total number of CCC and CCI. Specifically, the CITL was 0.007 (0.008 for the total number of CCC and -0.0003 for CCI), and the calibration slope was 1.027 (1.006 for the total number of CCC and 0.937 for CCI) (Table 4).

**Table 4.** Calibration of Children Comorbidity Score and Reference Models.

		Calibration in the large index (95% CI)	Calibration slope (95% CI)
Developed model	Children Comorbidity Score	0.007 (-0.053-0.066)	1.027 (0.981-1.073)
Reference models	Total number of ICD-10-defined CCC	0.008 (-0.050-0.067)	1.006 (0.953-1.060)
	Charlson Comorbidity Index	-0.0003 (-0.061-0.060)	0.937 (0.844-1.031)

ICD-10, International Classification of Diseases, 10th revision; CCC, complex chronic conditions; CI, confidence interval

Calibration plots are shown in Supplementary Figure 2. Whereas calibration plots are ordinarily depicted based on each of the ten deciles of predicted mortality groups, the plots shown here include only two groups. This is because the population included had an overall low mortality rate, and most of the predicted mortality values were concentrated in the lower range within the CCS and two reference models, resulting in identical values across the 10th and 90th percentiles within each model (Supplementary Table 6). Thus, the nine deciles were combined, and the entire population was divided into only two groups in the calibration plots.

**Figure 4.**
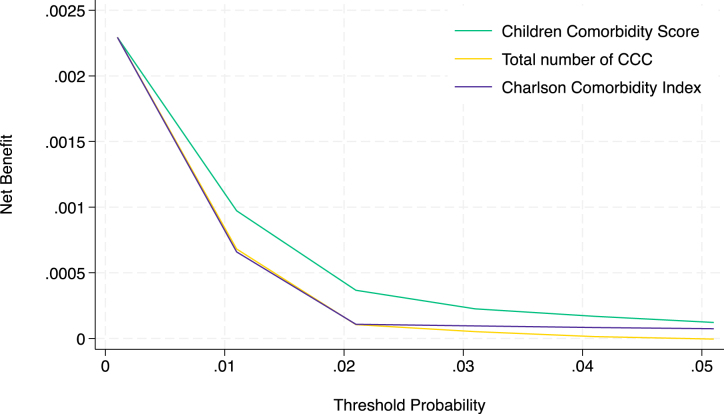
Decision curve analysis of the developed model (Children Comorbidity Score) and reference models for predicting in-hospital mortality.

In the clinical effectiveness evaluation using the decision curve analysis, the prediction using the CCS provided maximal benefit for preparation for in-hospital mortality compared with the prediction using the total number of CCC and CCI (Figure 4). The decision curve, including the two extreme default scenarios, is shown in Supplementary Figure 3.

### Stratified analysis and sensitivity analysis

The stratified analyses showed that the discrimination ability of the CCS, developed in the main analysis, for predicting in-hospital mortality remained superior to that of the total number of CCC and CCI across all age, sex, and BMI categories (Table 5).

**Table 5.** Discrimination Ability of the Children Comorbidity Score, Total Number of ICD-10-Defined CCC, and Charlson Comorbidity Index in the Subgroup Analyses.

				C-statistics (95% CI)	*p*-value
Age	3-5 years	Developed model	Children Comorbidity Score	0.744 (0.715-0.772)	Ref
		Reference models	Total number of ICD-10-defined CCC	0.660 (0.633-0.688)	< 0.001
			Charlson Comorbidity Index	0.534 (0.510-0.558)	< 0.001
	6-11 years	Developed model	Children Comorbidity Score	0.749 (0.724-0.775)	Ref
		Reference models	Total number of ICD-10-defined CCC	0.683 (0.657-0.708)	< 0.001
			Charlson Comorbidity Index	0.553 (0.531-0.574)	< 0.001
	12-17 years	Developed model	Children Comorbidity Score	0.681 (0.661-0.701)	Ref
		Reference models	Total number of ICD-10-defined CCC	0.622 (0.604-0.640)	< 0.001
			Charlson Comorbidity Index	0.553 (0.538-0.567)	< 0.001
Sex	male	Developed model	Children Comorbidity Score	0.711 (0.693-0.728)	Ref
		Reference models	Total number of ICD-10-defined CCC	0.643 (0.627-0.660)	< 0.001
			Charlson Comorbidity Index	0.541 (0.527-0.554)	< 0.001
	female	Developed model	Children Comorbidity Score	0.735 (0.713-0.757)	Ref
		Reference models	Total number of ICD-10-defined CCC	0.657 (0.637-0.678)	< 0.001
			Charlson Comorbidity Index	0.550 (0.532-0.568)	< 0.001
BMI	Underweight	Developed model	Children Comorbidity Score	0.810 (0.784-0.837)	Ref
		Reference models	Total number of ICD-10-defined CCC	0.725 (0.697-0.753)	< 0.001
			Charlson Comorbidity Index	0.607 (0.580-0.634)	< 0.001
	Normal	Developed model	Children Comorbidity Score	0.759 (0.735-0.783)	Ref
		Reference models	Total number of ICD-10-defined CCC	0.652 (0.629-0.675)	< 0.001
			Charlson Comorbidity Index	0.555 (0.535-0.575)	< 0.001
	Overweight and obesity	Developed model	Children Comorbidity Score	0.780 (0.735-0.826)	Ref
		Reference models	Total number of ICD-10-defined CCC	0.706 (0.661-0.752)	< 0.001
			Charlson Comorbidity Index	0.587 (0.545-0.629)	< 0.001

ICD-10, International Classification of Diseases, 10th revision; CCC, complex chronic conditions; CI, confidence interval; BMI, body mass index

In the sensitivity analysis, the CCS had better discrimination ability (C-statistics, 0.720 [0.707-0.734]) than the model using CCC categories fitted with logistic regression coefficients (0.651 [0.638-0.664], p < 0.001).

## Discussion

Using a nationwide inpatient database including 1,968,960 pediatric patients, we developed the CCS, an integer-based risk score based on comorbidities, to predict pediatric in-hospital mortality. The predictive ability of the CCS was better than that of the total number of CCC and the CCI. The CCS comprises 10 comorbidity categories identified using ICD-10 codes available at the time of admission.

Among patients who died during hospitalization (0.33%), comorbidities associated with mortality included neurological and neuromuscular disorders, cardiovascular conditions, gastrointestinal disease, hematologic and immunologic disorders, other congenital and genetic defects, malignancies, premature and neonatal disorders, use of medical devices, and transplantation. Among these comorbidities, malignancies, neurological and neuromuscular disorders, and cardiovascular conditions have been previously reported to be associated with one-year mortality after hospital discharge in pediatric patients ^(37)^. Comorbid malignancy and epilepsy or convulsions, categorized as neurological and neuromuscular disorders in our study, were also known to be strongly associated with the one-year risk of pediatric hospitalization ^(20)^. In both the CCI and prediction model for one-year prognosis in children ^(37)^, malignancy showed the strongest association with poor outcomes. Consistent with previous reports, our findings indicated that malignancy had the highest regression coefficient, followed by neurological and neuromuscular disorders, hematologic and immunologic conditions, cardiovascular diseases, and transplantation.

A factor associated with a decreased risk of in-hospital mortality was respiratory comorbidity. The respiratory disease category was incorporated into the CCS with a negative coefficient. The EC incorporates negative coefficients for several comorbidities, resulting in negative scores for those comorbidities. Similarly, comorbid respiratory diseases at admission were assigned negative values to predict postoperative pediatric complications ^(38)^. Such a negative coefficient, suggesting a decreased risk of mortality, may be attributed to potential coding bias, where the overall severity of a patient’s illness inversely affects the likelihood of coding non-threatening conditions ^(5), (8), (38)^. Consequently, the presence of codes for non-threatening diseases can indicate relatively healthy patients with a lower risk of in-hospital mortality.

The predictive ability of the CCS was similar to that of the CCI among adult patients. In this study, the C-statistics for the CCS in predicting in-hospital mortality among pediatric populations was 0.720 (95% CI, 0.707-0.734), which may be clinically useful given that the CCI, used worldwide as a weighted comorbidity index, had a C-statistics of 0.690 [95% CI, 0.688-0.692] among adult patients in a validation study using the Diagnosis Procedure Combination database ^(39)^, the same database used in our study. Therefore, the CCS in pediatric populations is expected to be as helpful as the CCI in adult populations for identifying high-risk patients. Given the widespread use of the CCI in adult database studies, the CCS is also anticipated to become widely used for risk adjustment in pediatric database studies.

Although we analyzed data from 1,625 hospitals across all prefectures in Japan, the distribution of average in-hospital mortality rates was highly right-skewed. Specifically, 59% of the 1,625 hospitals had an average in-hospital mortality rate of zero, and fewer than 5% had a rate exceeding 0.01. This skewness limited our ability to perform analyses and validations that account for hospital clustering. To maintain internal and external validity, we randomly split the population in a 4:1 ratio to create prediction and validation datasets. Additionally, 5-fold cross-validation was applied within the prediction set to facilitate comorbidity selection using Lasso regression. Despite these efforts to enhance the model’s robustness, further studies are necessary to validate the CCS model.

This study had several strengths and implications. First, we developed a comorbidity score for pediatric patients using comorbidities identified using ICD-10 codes, which are readily available at the time of admission in many countries. Therefore, we believe that this score has broad applicability. Second, we considered the weights of each comorbidity category and highlighted the importance of each, similar to the CCI and EC. We utilized a Lasso regression model to construct a parsimonious, efficient, and interpretable model. Recently, interpretable and transparent machine learning methods have gained attention alongside the acknowledged merits and demerits of black-box machine learning ^(40)^. Third, we achieved variable shrinkage using the 1SE rule of Lasso regression while maintaining discriminative value comparable to the model using all 56 variables (C-statistics, 0.733 [95% CI, 0.719-0.747]). Finally, we achieved better predictive ability in identifying patients who died during hospitalization using fewer variables while maintaining discrimination capabilities compared with conventional logistic regression models utilizing ICD-10-defined CCC, irrespective of its usage (weighted or total number of CCC).

This study had several limitations. First, we could not directly compare the predictive ability of the current model with that of the ICD-10-CM-defined CCC used in the United States because Japan does not adopt the ICD-10-CM codes; thus, we could not calculate the original pediatric CCC. Instead, we used the ICD-10-defined CCC as a reference model. Second, although CCS may be useful as a research tool for consistent comorbidity evaluation, particularly in studies with limited sample sizes or datasets lacking detailed clinical information, its effectiveness in frontline clinical decision-making should be assessed in future studies. In addition, the generalizability of the risk score is limited because we used only hospitalization data from Japan. Further studies are needed to confirm the validity of the score in countries outside Japan. Third, although the Diagnosis Procedure Combination database has been validated ^(23)^, some misclassifications may still be present. Fourth, this study did not include children aged 0-2 years, as neonates and infants require different predictors due to factors such as prematurity and perinatal conditions. Including this age group would hinder the development of a single, simplified comorbidity score applicable across all pediatric age groups. Instead, we focused on young children and adolescents aged 3-17 years. Further research is needed to assess the applicability of the CCS to children under 3 years of age.

In conclusion, using a nationwide Japanese inpatient database, we developed an integer-based CCS for predicting the risk of pediatric in-hospital mortality. We believe that this score is beneficial for risk assessment in database studies, which may help accumulate evidence in pediatric populations. Future validation studies of the integer-based score should be conducted in real clinical settings using different population databases.

## Article Information

### Conflicts of Interest

Kayo Ikeda Kurakawa, Akira Okada, Satoko Yamaguchi, and Takashi Kadowaki are members of the Department of Prevention of Diabetes and Lifestyle-Related Diseases, a cooperative program between The University of Tokyo and the Asahi Mutual Life Insurance Company. Kayo Ikeda Kurakawa was previously employed by Asahi Mutual Life Insurance Company. Takaaki Konishi, Nobuaki Michihata, Miho Ishimaru, Hiroki Matsui, Kiyohide Fushimi, Hideo Yasunaga, Toshimasa Yamauchi, and Masaomi Nangaku declare no conflicts of interest relevant to this article.

### Sources of Funding

This work was supported by the Ministry of Health, Labor and Welfare, Japan [grant numbers 23AA2003 and 22AA2003].

### Author Contributions

Study design: Kayo Ikeda Kurakawa, Akira Okada, Takaaki Konishi, and Nobuaki Michihata; data acquisition: Miho Ishimaru, Hiroki Matsui, and Kiyohide Fushimi; statistical analyses: Kayo Ikeda Kurakawa, Akira Okada, and Nobuaki Michihata; data interpretation: Kayo Ikeda Kurakawa, Akira Okada, Satoko Yamaguchi, Masaomi Nangaku, Toshimasa Yamauchi, Hideo Yasunaga, Takashi Kadowaki; supervision: Satoko Yamaguchi, Masaomi Nangaku, Toshimasa Yamauchi, Hideo Yasunaga, Takashi Kadowaki; funding acquisition: Hideo Yasunaga. Kayo Ikeda Kurakawa and Akira Okada contributed equally to this work.

### Approval by Institutional Review Board (IRB)

The Institutional Review Board of the University of Tokyo approved this study [approval number: 2018030NI]

### Informed Consent

As the study relied on anonymized retrospective data, the requirement for informed consent from individual patients was waived.

### Data Availability

The datasets analyzed during the current study are not publicly available due to contracts with the hospitals that provide data to the database.

### Current Address

Kayo Ikeda Kurakawa: Department of Pediatrics, National Rehabilitation Center for Persons with Disabilities, Namiki, Japan

## Supplement

Supplementary File 1 Supplementary Methods and Supplementary Tables.

Supplementary File 2 Children Comorbidity Score Stata Program.

Supplementary Figures Supplementary Figure 1　Histogram of the average in-hospital mortality rate per hospital. Histogram of the average in-hospital mortality rate per hospital. For clarity, this figure includes data up to the 99th percentile of the total number of hospitals.
Supplementary Figure 2　Calibration plots of the Children Comorbidity Score and reference models for predicting in-hospital mortality. Due to the low probability of in-hospital mortality, we presented a plot limited to the display range of predicted probabilities up to 0.1.
Supplementary Figure 3　Decision curve analysis of the developed model (Children Comorbidity Score) and reference models for predicting in-hospital mortality including two extreme default scenarios.
The “treat none” line represents a scenario where no patients receive preventive care for the risk of in-hospital death, avoiding unnecessary interventions but failing to support any high-risk patients, resulting in a net benefit of zero across all risk thresholds. On the other hand, the “treat all” line represents a scenario where all patients are prepared for the risk of in-hospital death, regardless of their risk, ensuring no high-risk cases are missed but leading to unnecessary interventions for low-risk patients.
