# Supplementary material for: Children Comorbidity Score, a Simple Predictor for In-hospital Mortality: A Nationwide Inpatient Database Study in Japan
Source: JMA J. 2025 Apr 4;8(2):568–79. doi: 10.31662/jmaj.2024-0333 (PMC12095624; doi:10.31662/jmaj.2024-0333)
Supplement: Supplementary File 1 — Supplementary Methods and Supplementary Tables. [file 2433-3298-8-2-0568-s001.pdf]

Supplementary File 1

Supplementary Methods

Preparation of 12 primary comorbidity categories and 44 comorbid subcategories belonging to 12 primary comorbidity categories

Overview:

Step 1: Prepare 12 tentative primary comorbidity categories based on the Pediatric Complex Chronic Conditions classification system.

Step 2: Recategorize ICD-10 codes regarding (sub)categories based on the Pediatric Complex Chronic Conditions classification system, Charlson, and Elixhauser indices into compacted subcategories within the primary comorbidity category.

Step 3: Incorporate the ICD-10 codes, not included in the subcategories in Step 2 but ranked top 100 among patients undergoing in-hospital mortality, into the primary comorbidity category.

Step 1: Prepare 12 tentative primary comorbidity categories based on the Pediatric Complex Chronic Conditions classification system.

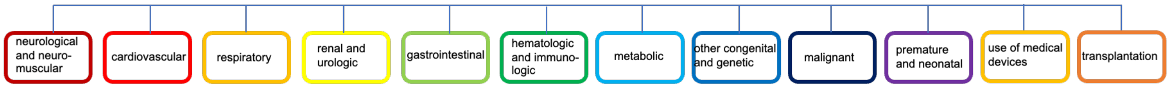

Step 2: Recategorize ICD-10 codes regarding (sub)categories based on the Pediatric Complex Chronic Conditions classification system, Charlson, and Elixhauser indices into compacted subcategories within the primary comorbidity category.

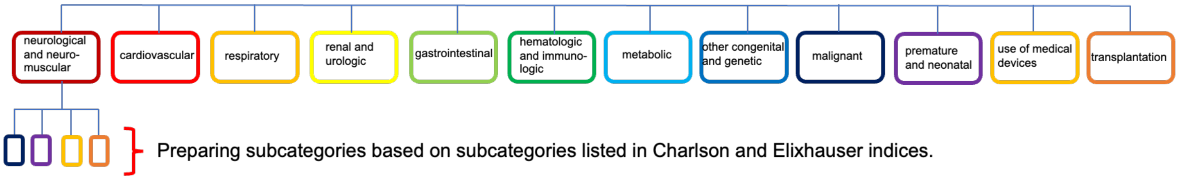

Step 3: Incorporate the ICD-10 codes, not included in the subcategories in Step 2 but ranked top 100 among patients undergoing in-hospital mortality, into the primary comorbidity category.

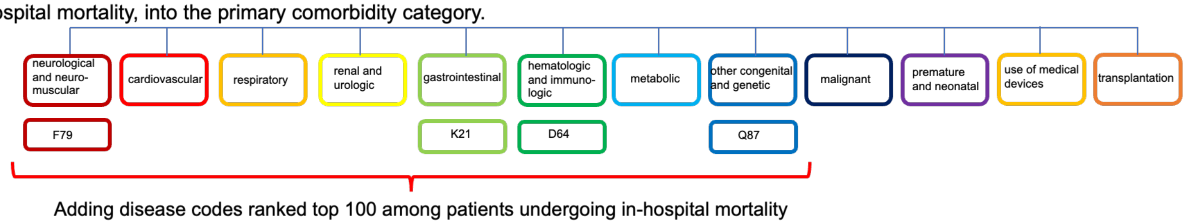

Example: The process of creating a primary comorbidity category (gastrointestinal) and its four subcategories (peptic ulcer, gastrointestinal disease, mild liver disease, moderate or severe liver disease) included in the primary comorbidity category.

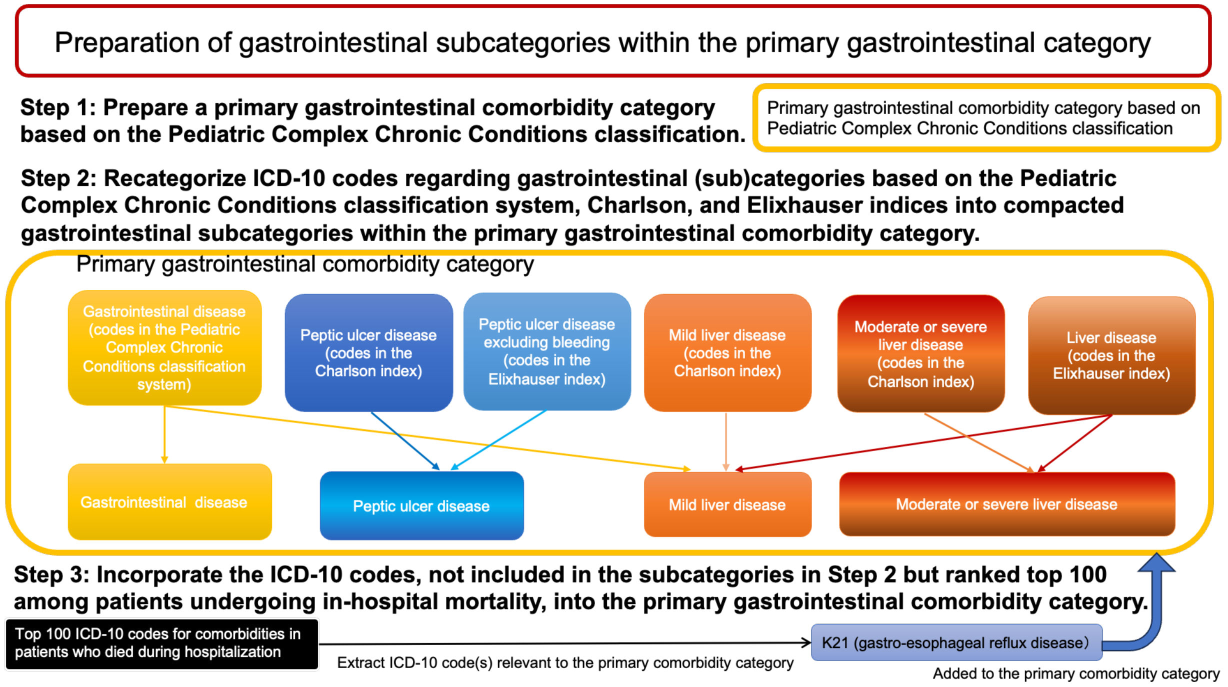

Step 1: Prepare a primary gastrointestinal comorbidity category based on the Pediatric Complex Chronic Conditions classification.

Gastrointestinal disease: K50 K51 K73 K74 Q41 Q42 Q43 Q44 Q45 K754 K760 K761 K762 K763 K768 Q390 Q391 Q392 Q393 Q394 I820 K551 K562 K593 Z980 Z903 Z944 Z931 Z932 Z933 Z934 Z431 Z432

Step 2: Recategorize ICD-10 codes regarding gastrointestinal (sub)categories based on the Pediatric Complex Chronic Conditions classification system, Charlson, and Elixhauser indices into compacted gastrointestinal subcategories within the primary comorbidity category for gastrointestinal disease.

The ICD-10 codes in "peptic ulcer disease excluding bleeding" in the Elixhauser index were almost identical to those listed under "peptic ulcer disease" in the Charlson index. Therefore, the "peptic ulcer disease" category was adopted in the gastrointestinal subcategories.

Apart from K720, the ICD-10 codes in "Liver disease" category in the Elixhauser index were encompassed within the Charlson index categories "mild liver disease" or "moderate or severe liver disease." Therefore, we introduced K720 into the "moderate or severe liver disease" category, while the Charlson index category "mild liver disease" was used without changes.

From the gastrointestinal disease category within the Pediatric Complex Chronic Conditions classification system, we removed the codes that were also included in the Charlson index and Elixhauser index.

Original gastrointestinal (sub)categories in ICD-10 defined Pediatric Complex Chronic Conditions classification system, Charlson, and Elixhauser indices

Pediatric Complex Chronic Conditions classification **(Original)**:
Gastrointestinal disease: K50 K51 K73 K74 Q41 Q42 Q43 Q44 Q45 K754 K760 K761 K762 K763 K768 Q390 Q391 Q392 Q393 Q394 I820 K551 K562 K593 Z980 Z903 Z944 Z931 Z932 Z933 Z934 Z431 Z432
Charlson comorbidity index **(Original)**:
Mild liver disease: B18 K73 K74 K700 K701 K702 K703 K709 K713 K714 K715 K717 K760 K762 K763 K764 K768 K769 Z944
Moderate or severe liver disease: I85 I864 I982 K704 K711 K721 K729 K765 K766 K767
Peptic ulcer disease: K25 K26 K27 K28
Elixhauser comorbidity index **(Original)**:
Liver disease: B18 I85 I864 I982 K70 K711 K713 K714 K715 K717 K72 K73 K74 K760 K762 K763 K764 K765 K766 K767 K768 K769 Z944
Peptic ulcer disease excluding bleeding: K257 K259 K267 K269 K277 K279 K287 K289

Through the process described previously, four subcategories of gastrointestinal were prepared.

Four subcategories within the primary gastrointestinal category **(Newly prepared)**

1. Peptic ulcer disease (Identical to the CCI classification): K25 K26 K27 K28
2. Mild liver disease (Identical to the CCI classification): B18 K73 K74 K700 K701 K702 K703 K709 K713 K714 K715 K717 K760 K762 K763 K764 K768 K769 Z944
3. Moderate or severe liver disease (recategorized): I85 I864 I982 K704 K711 K720 K721 K729 K765 K766 K767
4. Gastrointestinal disease (recategorized): K50 K51 Q41 Q42 Q43 Q44 Q45 K754 K761 Q390 Q391 Q392 Q393 Q394

Step 3: Incorporate the ICD-10 codes, not included in the subcategories in Step 2 but ranked top 100 among patients undergoing in-hospital mortality, into the primary gastrointestinal comorbidity category.

Among the top 100 ICD-10 codes for comorbidities in patients who died during hospitalization, K21 (gastroesophageal reflux disease), which was not covered by the Pediatric Complex Chronic Conditions classification system, Charlson, or Elixhauser indices, was added to four gastrointestinal subcategories to finalize the gastrointestinal category within the 12 primary comorbidity categories.

Finalized primary gastrointestinal comorbidity category: B18 I820 I85 I864 I982 K21 K25 K26 K27 K28 K50 K51 K551 K562 K593 K70 K711 K713 K714 K715 K717 K72 K73 K74 K754 K760 K761 K762 K763 K764 K765 K766 K767 K768 K769 Q390 Q391 Q392 Q393 Q394 Q41 Q42 Q43 Q44 Q45 Z431 Z432 Z433 Z434 Z903 Z931 Z932 Z933 Z934 Z944 Z980

Supplementary Tables

Supplementary Table 1. Forty-four comorbid subcategories

Comorbid categories	ICD-10 codes
**Neurological and neuromuscular**	
Neuromuscular disease	E750-E752 E754 F71-F73 F842 G230-G232 G238 G248 G253 G259 G71 G72 G901 G909 G911 G935 G938 G939 G94 R403 Q00-Q07 Q851 Z982
Hemiplegia and paralysis	G041 G114 G80-G82 G830-G835 G839
Other neurological disorders	G10-G13 G20-G22 G254 G255 G312 G318 G319 G32 G35-G37 G40 G41 G931 G934 R470 R56
**Cardiovascular**	
Acute myocardial infarction	I21 I22 I252
Cardiac arrhythmia	I44 I45 I47-I49 R000 R001 R008 T821 Z450 Z950
Congestive heart failure	I099 I110 I130 I132 I255 I420 I425 I426 I427 I428 I429 I43 I50 P290
Valvular disease	A520 I05-I08 I091 I098 I34-I39 Q230-Q233 Z952-Z954
Pulmonary circulation disorders	I26 I27 I280 I288 I289
Cerebrovascular disease	G45 G46 H340 I6
Peripheral vascular disease	I70 I71 I731 I738 I739 I771 I790 I792 K551 K558 K559 Z958 Z959
Hypertension, uncomplicated	I10
Hypertension, complicated	I11-I13 I15
Other cardiovascular disease	I421-I424 I515 I517 Q212-Q214 Q218 Q219 Q22 Q234-Q239 Q24 Q251-Q259 Q26 Q282 Q283 Q289 Z941 Z951
**Respiratory**	
Respiratory disease	E84 I43 P280 Q30-Q34 Z430 Z902 Z930 Z942 Z990

Chronic pulmonary disease	I278 I279 J4 J60-J67 J684 J701 J703
**Renal and urologic**	
Renal disease other than renal failure	G834 N032-N037 N052-N057 N312 N319 Q60-Q64 Z435 Z436 Z446 Z905 Z906 Z936
Renal failure	I120 I131 N18 N19 N250 Z490-Z492 Z940 Z992
**Gastrointestinal**	
Peptic ulcer	K25-K28
Gastrointestinal disease	I820 K50 K51 K551 K562 K593 K754 K761 Q390-Q394 Q41-Q45 Z431-Z434 Z980 Z903 Z931-Z934
Mild liver disease	B18 K700-K703 K709 K713-K715 K717 K73 K74 K760 K762-K764 K768 K769 Z944
Moderate or severe liver disease	I85 I864 I982 K704 K711 K720 K721 K729 K765 K766 K767
**Hematologic and immunologic**	
Coagulopathy	D65-D68 D691 D693-D696
Blood loss anemia	D500
Deficiency anemia	D508 D509 D51-D53
Human immunodeficiency virus disease	B20 B21 B23 B24
Collagen disease	L940 L941 L943 M05 M06 M08 M120 M123 M30 M310-M313 M315 M32-M35 M360 M45 M461 M468 M469
Other hematologic disorders, immunodeficiencies, and related conditions	D55-D58 D60 D61 D700 D704 D71 D720 D761-D763 D80-D88 M314 M316 Z21
**Metabolic**	
Metabolic disease	D841 E230 E232 E233 E237 E240 E242 E243 E248-E250 E258 E259 E700 E702-E705 E708 E710-E715 E720-E724 E728 E729 E740-E744 E748 E749 E75 E760-E763 E770 E771 E780-E789 E791 E798 E804-E807 E830 E831 E833 E834 E85 E88 H498 Z794
Obesity	E66

Diabetes Uncomplicated	E100 E101 E109-E111 E119-E121 E129-E131 E139-E141 E149
Diabetes Complicated	E102-E108 E112-E118 E122-E128 E132-E138 E142-E148
Hypothyroidism	E00-E03 E890
Weight loss	E40-E46 R634 R64
Fluid loss and electrolyte disorders	E222 E86 E87
**Other congenital or genetic defect**	
Other congenital or genetic defect	E343 K449 M410 M412 M418 M419 M965 Q722 Q750 Q752 Q759 Q760-Q762 Q764-Q767 Q77 Q780-Q784 Q788-Q795 Q799 Q81 Q897-Q899 Q909 Q913 Q914 Q917 Q928 Q93 Q950 Q969 Q97 Q98 Q992 Q998 Q999
**Malignancy**	
Solid tumor without metastasis	C00-C26 C30-C34 C37-C41 C43 C45-C58 C60-C76 C97
Metastatic cancer	C77-C80
Leukemia and lymphoma	C81-C85 C88 C90-C96
Tumors	D00-D09 D37-D49 Q850
**Others**	
Dementia	F00-F03 F051 G30 G311
Alcohol abuse	E52 F10 G621 I426 K292 K700 K703 K709 T51 Z502 Z714 Z721
Drug abuse	F11-F16 F18 F19 Z715 Z722
Depression	F204 F313-F315 F32 F33 F341 F412 F432
Psychoses	F20 F22-F25 F28 F29 F302 F312 F315

ICD-10, International Classification of Diseases, 10th revision; CCC, complex chronic conditions.

Supplementary Table 2. Ranking of ICD-10 codes for comorbidities at admission among patients who died during hospitalization.

Rank	ICD-10 code	Number of patients with the ICD-10 code	Addition of ICD-10 codes for the 12 primary comorbidity categories	ICD-10-defined CCC	CCI	EC
1	J96 (Respiratory failure, not elsewhere classified)	721 (11.1%)				
2	G40 (Epilepsy)	625 (9.63%)				Covered
3	I46 (Cardiac arrest)	482 (7.42%)				
4	G93 (Other disorders of brain)	481 (7.41%)		Partially covered		Partially covered
5	R57 (Shock, not elsewhere classified)	409 (6.3%)				
6	I50 (Heart failure)	339 (5.22%)		Partially covered	Covered	Covered
7	S27 (Injury of other and unspecified intrathoracic organs)	292 (4.5%)				
8	S06 (Intracranial injury)	285(4.39%)				
9	S02 (Fracture of skull and facial bones)	234 (3.6%)				
10	D65 (Disseminated intravascular coagulation [defibrination syndrome])	228 (3.51%)				Covered
11	G80 (Cerebral palsy)	212 (3.27%)				Partially covered

12	D69 (Purpura and other hemorrhagic conditions)	211 (3.25%)		Covered	Partially covered	Partially covered
13	R40 (Somnolence, stupor, and coma)	208 (3.2%)		Partially covered		
14	E87 (Other disorders of fluid, electrolyte, and acid-base balance)	183 (2.82%)				Covered
15	G91 (Hydrocephalus)	172 (2.65%)		Partially covered		
16	J18 (Pneumonia, unspecified organism)	171 (2.63%)				
17	A41 (Other sepsis)	163 (2.51%)				
17	R52 (Pain, not elsewhere classified)	163 (2.51%)				
19	D64 (Other anemias)	154 (2.37%)	Yes			
20	J69 (Pneumonitis due to solids and liquids)	151 (2.33%)				
21	S32 (Fracture of lumbar spine and pelvis)	143 (2.2%)				
22	C79 (Secondary malignant neoplasm of other and unspecified sites)	142 (2.19%)		Covered	Covered	Covered
23	J45 (Asthma)	132(2.03%)			Covered	Covered
24	Z93 (Artificial opening status)	127(1.96%)		Partially covered		

25	C78 (secondary malignant neoplasm of respiratory and digestive organs)	123 (1.89%)	Covered	Covered	Covered
26	E23 (Hypofunction and other disorders of pituitary gland)	119 (1.83%)	Partially covered		
27	T79 (Certain early complications of trauma, not elsewhere classified)	114 (1.76%)			
28	R09 (Other symptoms and signs involving the circulatory and respiratory systems)	111 (1.71%)			
29	Q21 (Congenital malformations of cardiac septa)	110 (1.69%)	Partially covered		
30	K25 (Gastric ulcer)	107 (1.65%)		Covered	Partially covered
31	B59 (Pneumocystosis)	99 (1.52%)			
32	S36 (Injury of intra-abdominal organs)	99 (1.52%)			
33	N17 (Acute renal failure)	94 (1.45%)			
34	I49 (Other cardiac arrhythmias)	94 (1.45%)	Partially covered		Covered
35	T86 (Failure and rejection of transplanted organs and tissues)	94 (1.45%)	Partially covered		

36	T75 (Effects of other external causes)	93 (1.43%)				
37	K21 (Gastro-esophageal reflux disease)	91 (1.4%)	Yes			
38	Z94 (Transplanted organ and tissue status)	91 (1.4%)		Partially covered	Partially covered	Partially covered
39	B48 (Other mycoses, not elsewhere classified)	87 (1.34%)				
40	I27 (Other pulmonary heart diseases)	87 (1.34%)		Partially covered	Partially covered	Covered
41	D50 (Iron deficiency anemia)	83 (1.28%)				Partially covered
42	E86 (Volume depletion)	81 (1.25%)				Covered
43	S72 (Fracture of femur)	79 (1.22%)				
44	K59 (Other functional intestinal disorders)	79 (1.22%)		Partially covered		
45	E88 (Other metabolic disorders)	77 (1.19%)		Covered		
46	G71 (Primary disorders of muscles)	76 (1.17%)		Covered		
47	I10 (Essential (primary) hypertension)	73 (1.12%)				Covered
48	R63 (Symptoms and signs concerning food and fluid intake)	71 (1.09%)				Partially covered
49	S22 (Fracture of rib(s), sternum and thoracic spine)	69 (1.06%)				

50	E03 (Other hypothyroidism)	68 (1.05%)			Covered
51	R11 (Nausea and vomiting)	68 (1.05%)			
52	R56 (Convulsions, not elsewhere classified)	68 (1.05%)			Covered
53	I42 (Cardiomyopathy)	67 (1.03%)	Covered	Partially covered	Partially covered
54	R68 (other general symptoms and signs)	63 (0.97%)			
55	Q25 (Congenital malformations of great arteries)	63 (0.97%)	Partially covered		
56	Q04 (Other congenital malformations of brain)	62 (0.95%)	Covered		
57	I61 (Intracerebral hemorrhage)	62 (0.95%)		Covered	
58	D80 (Immunodeficiency with predominantly antibody defects)	61 (0.94%)	Covered		
58	J98 (Other respiratory disorders)	61 (0.94%)			
58	D68 (Other coagulation defects)	61 (0.94%)	Partially covered		Covered
58	D70 (Agranulocytosis)	61 (0.94%)	Partially covered		
62	R13 (Dysphagia)	59 (0.91%)			
62	J81 (Pulmonary oedema)	59 (0.91%)			
62	K76 (Other diseases of liver)	59 (0.91%)	Partially covered	Partially covered	Partially covered

65	C71 (Malignant neoplasm of brain)	58 (0.89%)	Covered	Covered	Covered
66	J15 (Bacterial pneumonia, not elsewhere classified)	56 (0.86%)			
67	S01 (Open wound of head)	55 (0.85%)			
68	Q91 (Edwards syndrome and Patau syndrome)	53 (0.82%)	Partially covered		
69	M62 (Other disorders of muscle)	49 (0.75%)			
69	K92 (Other diseases of digestive system)	49 (0.75%)			
71	E79 (Disorders of purine and pyrimidine metabolism)	48 (0.74%)	Partially covered		
71	Q90 (Down syndrome)	48 (0.74%)	Partially covered		
71	A09 (Other gastroenteritis and colitis of infectious and unspecified origin)	48 (0.74%)			
74	E83 (Disorders of mineral metabolism)	47 (0.72%)	Partially covered		
75	T14 (Injury of unspecified body region)	46 (0.71%)			
75	Q20 (Congenital malformations of cardiac chambers and connections)	46 (0.71%)	Covered		
75	S42 (Fracture of shoulder and upper arm)	46 (0.71%)			

75	S09 (Other and unspecified injuries of head)	46 (0.71%)				
79	J90 (Pleural effusion, not elsewhere classified)	44 (0.68%)				
79	A49 (Bacterial infection of unspecified site)	44 (0.68%)				
79	K56 (Paralytic ileus and intestinal obstruction without hernia)	44 (0.68%)		Partially covered		
82	T71 (Asphyxiation)	43 (0.66%)				
83	T07 (Unspecified multiple injuries)	40 (0.62%)				
83	G82 (Paraplegia and tetraplegia)	40 (0.62%)			Covered	Covered
85	S82 (Fracture of lower leg, including ankle)	38 (0.59%)				
85	J80 (Adult respiratory distress syndrome)	38 (0.59%)				
87	Q87 (Other specified congenital malformation syndromes affecting multiple systems)	37 (0.57%)	Yes			
87	N18 (Chronic kidney disease)	37 (0.57%)		Covered	Covered	Covered
89	J42 (Unspecified chronic bronchitis)	36 (0.55%)			Covered	Covered
89	R04 (Hemorrhage from respiratory passages)	36 (0.55%)				

89	N39 (Other disorders of urinary system)	36 (0.55%)				
92	F79 (Unspecified mental retardation)	34 (0.52%)	Yes			
93	T00 (Superficial injuries involving multiple body regions)	33 (0.51%)				
93	K72 (Hepatic failure, not elsewhere classified)	33 (0.51%)			Partially covered	Covered
93	D43 (Neoplasm of uncertain or unknown behavior of brain and central nervous system)	33 (0.51%)		Covered		
96	J20 (Acute bronchitis)	31 (0.48%)				
97	Q89 (Other congenital malformations, not elsewhere classified)	30(0.46%)		Partially covered		
97	F32 (Depressive episode)	30 (0.46%)				Covered
97	E71 (Disorders of branched-chain amino-acid metabolism and fatty-acid metabolism)	30 (0.46%)		Partially covered		
100	F84 (Pervasive developmental disorders)	29 (0.45%)		Partially covered		
100	B37 (Candidiasis)	29 (0.45%)				
100	K29 (Gastritis and duodenitis)	29 (0.45%)				Partially covered

Blank indicates not covered. Disease codes that were not included in pediatric CCC, the Charlson Comorbidity Index, or the Elixhauser Comorbidity Index, but still reflected an acute condition, were not added to the 12 primary comorbidity categories.

ICD-10, International Classification of Diseases, 10th revision; CCC, complex chronic conditions; CCI, Charlson Comorbidity Index; EC, Elixhauser Comorbidity Index.

Supplementary Table 3. ICD-10 codes of the 12 primary comorbidity categories

Categories	ICD-10 codes
Neurological and neuromuscular	E750 E751 E752 E754 F71 F72 F73 F79 F842 G041 G10 G11 G12 G13 G20 G21 G22 G230 G231 G232 G238 G248 G253 G254 G255 G259 G312 G318 G319 G32 G35 G36 G37 G40 G41 G71 G72 G80 G81 G82 G830 G831 G832 G833 G834 G835 G839 G901 G909 G911 G931 G934 G935 G938 G939 G94 Q00 Q01 Q02 Q03 Q04 Q05 Q06 Q07 Q851 R403 R470 R56 Z982
Cardiovascular	A520 G45 G46 H340 I05 I06 I07 I08 I091 I098 I099 I10 I11 I12 I13 I15 I21 I22 I252 I255 I26 I27 I280 I288 I289 I34 I35 I36 I37 I38 I39 I42 I43 I44 I45 I47 I48 I49 I50 I515 I517 I6 I70 I71 I731 I738 I739 I771 I790 I792 K551 K558 K559 P290 Q20 Q212 Q213 Q214 Q218 Q219 Q22 Q23 Q24 Q251 Q252 Q253 Q254 Q255 Q256 Q257 Q258 Q259 Q26 Q282 Q283 Q289 R000 R001 R008 T821 Z450 Z941 Z950 Z951 Z952 Z953 Z954 Z958 Z959
Respiratory	E84 I278 I279 I43 J4 J60 J61 J62 J63 J64 J65 J66 J67 J684 J701 J703 P280 Q30 Q31 Q32 Q33 Q34 Z430 Z902 Z930 Z942 Z990

Renal and urologic	G834 I120 I131 N032 N033 N034 N035 N036 N037 N052 N053 N054 N055 N056 N057 N18 N19 N250 N312 N319 Q60 Q61 Q62 Q63 Q64 Z435 Z436 Z446 Z490 Z491 Z492 Z905 Z906 Z936 Z940 Z992
Gastrointestinal	B18 I820 I85 I864 I982 K21 K25 K26 K27 K28 K50 K51 K551 K562 K593 K70 K711 K713 K714 K715 K717 K72 K73 K74 K754 K760 K761 K762 K763 K764 K765 K766 K767 K768 K769 Q390 Q391 Q392 Q393 Q394 Q41 Q42 Q43 Q44 Q45 Z431 Z432 Z433 Z434 Z903 Z931 Z932 Z933 Z934 Z944 Z980
Hematologic and immunologic	B20 B21 B22 B23 B24 D500 D508 D509 D51 D52 D53 D55 D56 D57 D58 D60 D61 D64 D65 D66 D67 D68 D691 D693 D694 D695 D696 D700 D704 D71 D720 D761 D762 D763 D80 D81 D82 D83 D84 D85 D86 D87 D88 L940 L941 L943 M05 M06 M08 M120 M123 M30 M310 M311 M312 M313 M314 M315 M316 M32 M33 M34 M35 M360 M45 M461 M468 M469 Z21
Metabolic	D841 E00 E009 E01 E02 E03 E100 E101 E102 E103 E104 E105 E106 E107 E108 E109 E110 E111 E112 E113 E114 E115 E116 E117 E118 E119 E120 E121 E122 E123 E124 E125 E126 E127 E128 E129 E130 E131 E132 E133 E134 E135 E136 E137 E138 E139 E140 E141 E142 E143 E144 E145 E146 E147 E148

	E149 E222 E230 E232 E233 E237 E240 E242 E243 E248 E249 E250 E258 E259 E40 E41 E42 E43 E44 E45 E46 E66 E700 E702 E703 E704 E705 E708 E710 E711 E712 E713 E714 E715 E720 E721 E722 E723 E724 E728 E729 E740 E741 E742 E743 E744 E748 E749 E75 E760 E761 E762 E763 E770 E771 E780 E781 E782 E783 E784 E785 E786 E787 E788 E789 E791 E798 E804 E805 E806 E807 E830 E831 E833 E834 E85 E86 E87 E88 E890 H498 R634 R64 Z794
Other congenital or genetic defect	E343 K449 M410 M412 M418 M419 M965 Q722 Q750 Q752 Q759 Q760 Q761 Q762 Q764 Q765 Q766 Q767 Q77 Q780 Q781 Q782 Q783 Q784 Q788 Q789 Q790 Q791 Q792 Q793 Q794 Q795 Q799 Q81 Q87 Q897 Q898 Q899 Q909 Q913 Q914 Q917 Q928 Q93 Q950 Q969 Q97 Q98 Q992 Q998 Q999
Malignancy	C D00 D01 D02 D03 D04 D05 D06 D07 D08 D09 D37 D38 D39 D40 D41 D42 D43 D44 D45 D46 D47 D48 D49 Q850
Premature and neonatal	P052 P059 P100 P101 P104 P115 P210 P219 P250 P251 P253 P258 P270 P271 P278 P350 P351 P524 P528 P560 P570 P578 P613 P614 P773 P832 P84 P912 P916

Use of medical devices	T865 T872 Y831 Y833 Z430 Z431 Z432 Z433 Z434 Z435 Z436 Z446 Z930 Z931 Z932 Z933 Z934 Z936 Z940 Z942 Z950 Z952 Z953 Z959 Z982 Z990 Z992
Transplantation	T86 Z94

ICD-10, International Classification of Diseases, 10th revision.

Supplementary Table 4. Categories of admission-precipitating diagnoses of the patients

Categories of admission-precipitating diagnosis	Hospital survival group (N=1,962,468)	In-hospital mortality group (N=6,492)	Total (N=1,968,960)
Infectious disease (ICD-10 codes: A, B)	158,223 (8.1%)	201 (3.1%)	158,424 (8.0%)
Malignancy (ICD-10 codes: C-D49)	73,337 (3.7%)	1,364 (21.0%)	74,701 (3.8%)
Blood disorders (ICD-10 codes: D50-99)	27,810 (1.4%)	121 (1.9%)	27,931 (1.4%)
Endocrine (ICD-10 codes: E)	77,692 (4.0%)	79 (1.2%)	77,771 (3.9%)
Mental disorders (ICD-10 codes: F)	18,233 (0.9%)	15 (0.2%)	18,248 (0.9%)
Neurological disorders (ICD-10 codes: G)	93,846 (4.8%)	386 (5.9%)	94,232 (4.8%)
Ophthalmological disorders(ICD-10 codes: H00-59)	46,675 (2.4%)	1 (0.0%)	46,676 (2.4%)
Otological disorders (ICD-10 codes: H60–H95)	44,851 (2.3%)	0 (0.0%)	44,851 (2.3%)
Cardiovascular (ICD-10 codes: I)	28,799 (1.5%)	2,254 (34.7%)	31,053 (1.6%)
Respiratory (ICD-10 codes: J)	483,399 (24.6%)	551 (8.5%)	483,950 (24.6%)
Digestive (ICD-10 codes: K)	199,028 (10.1%)	114 (1.8%)	199,142 (10.1%)
Dermatological (ICD-10 codes: L)	50,618 (2.6%)	8 (0.1%)	50,626 (2.6%)
Musculoskeletal (ICD-10 codes: M)	87,163 (4.4%)	24 (0.4%)	87,187 (4.4%)
Genitourinary (ICD-10 codes: N)	63,685 (3.2%)	41 (0.6%)	63,726 (3.2%)
Pregnancy (ICD-10 codes: O)	6,798 (0.3%)	0 (0.0%)	6,798 (0.3%)
Perinatal (ICD-10 codes: P)	555 (0.0%)	1 (0.0%)	556 (0.0%)
Congenital (ICD-10 codes: Q)	96,680 (4.9%)	157 (2.4%)	96,837 (4.9%)
Symptoms/signs (ICD-10 codes: R)	62,032 (3.2%)	191 (2.9%)	62,223 (3.2%)
Injury/Poisoning (ICD-10 codes: S, T)	341,937 (17.4%)	977 (15.0%)	342,914 (17.4%)
External causes (ICD-10 codes: V, W, X, Y)	12 (0.0%)	6 (0.1%)	18 (0.0%)
Examinations (ICD-10 codes: Z)	1,095 (0.1%)	1 (0.0%)	1,096 (0.1%)

ICD-10, International Classification of Diseases, 10th revision.

Supplementary Table 5. Clinical characteristics of patients assigned to the training or the test set

	Training set (N=1,575,168)		Test set (N=393,792)		Absolute Standardized difference
Sex (male)	906,480	(57.5%)	226,303	(57.5%)	0.002
Age (years)	8.92	4.64	8.91	4.64	0.003
Age (years)					
3-5	512,383	(32.5%)	128,196	(32.6%)	0.001
6-11	549,957	(34.9%)	137,861	(35.0%)	0.002
12-17	512,828	(32.6%)	127,735	(32.4%)	0.003
Body mass index					
Underweight	227,216	(14.4%)	56,619	(14.4%)	0.001
Normal	1,064,938	(67.6%)	266,169	(67.6%)	0.000
Overweight and obesity	139,868	(8.9%)	35,116	(8.9%)	0.001
Missing	143,146	(9.1%)	35,888	(9.1%)	0.001
Use of ambulance on admission					
No	1,417,224	(90.0%)	354,020	(89.9%)	0.002
Yes	157,405	(10.0%)	39,628	(10.1%)	0.002
Missing	539	(0.0%)	144	(0.0%)	0.001
Unscheduled admission					
No	730,560	(46.4%)	182,168	(46.3%)	0.002
Yes	844,055	(53.6%)	211,476	(53.7%)	0.002
Missing	553	(0.0%)	148	(0.0%)	0.001
Comorbidities					
Neurological and neuromuscular	50,925	(3.2%)	12,726	(3.2%)	0.000
Cardiovascular	30,991	(2.0%)	7,712	(2.0%)	0.001
Respiratory	116,914	(7.4%)	29,086	(7.4%)	0.001
Renal and urologic	4,531	(0.3%)	1,096	(0.3%)	0.002
Gastrointestinal	30,333	(1.9%)	7,434	(1.9%)	0.003
Hematologic and immunologic	21,104	(1.3%)	5,388	(1.4%)	0.002
Metabolic	116,546	(7.4%)	29,144	(7.4%)	0.000
Other congenital and genetic	12,074	(0.8%)	3,082	(0.8%)	0.002
Malignancy	8,486	(0.5%)	2,020	(0.5%)	0.004
Premature and neonatal	515	(0.0%)	130	(0.0%)	0.000
Use of medical devices	3,280	(0.2%)	742	(0.2%)	0.004
Transplantation	2,413	(0.2%)	542	(0.1%)	0.004
Charlson Comorbidity Index	0.110	(0.40)	0.110	(0.40)	0.002
Total number of CCC	0.054	(0.26)	0.053	(0.26)	0.002
In-hospital mortality	5,196	(0.3%)	1,296	(0.3%)	0.000

Data are presented as n (%) or mean (standard deviation).

CCC, complex chronic conditions

Supplementary Table 6. The distribution of scores and predicted in-hospital mortality for the Children Comorbidity Score and reference models

Children Comorbidity Score				Total number of pediatric complex chronic conditions				Charlson Comorbidity Index			
Score	Predicted probability	Number of individuals	Cumulative frequency	Score	Predicted probability	Number of individuals	Cumulative frequency	Score	Predicted probability	Number of individuals	Cumulative frequency
0	0.00103481	26,231	6.6611%	0	0.00262203	375,605	95.3816%	0	0.00282354	358,678	91.0831%
1	0.00132512	313	6.7406%	1	0.0100496	15,934	99.4279%	1	0.00552566	28,939	98.4319%
3	0.00217234	333,255	91.3678%	2	0.03772191	1,844	99.8961%	2	0.01078573	5,344	99.7890%
4	0.00278088	5,207	92.6900%	3	0.13147241	346	99.9840%	3	0.02094759	418	99.8951%
5	0.00355929	49	92.7025%	4	0.3688992	58	99.9987%	4	0.04029353	129	99.9279%
6	0.0045546	2,324	93.2927%	5	0.69298631	4	99.9997%	5	0.07611719	16	99.9319%
7	0.0058266	338	93.3785%	6	0.89707863	1	100.0000%	6	0.13917179	214	99.9863%
8	0.00745118	1,146	93.6695%					7	0.24084349	3	99.9870%
9	0.00952439	5,485	95.0624%					8	0.3836852	47	99.9990%
10	0.01216738	4,312	96.1574%					9	0.54988098	3	99.9997%
11	0.01553229	10,391	98.7961%					10	0.70564282	1	100.0000%
12	0.01980911	616	98.9525%								
13	0.02523336	1,873	99.4281%								
14	0.03209429	331	99.5122%								
15	0.04074273	48	99.5244%								
16	0.05159742	671	99.6948%								
17	0.06514762	537	99.8311%								
18	0.08194881	189	99.8791%								
19	0.10260735	113	99.9078%								
20	0.12774906	168	99.9505%								
21	0.15796676	117	99.9802%								
22	0.19374453	21	99.9855%								
23	0.23536056	8	99.9876%								
24	0.28278044	17	99.9919%								
25	0.3355616	3	99.9926%								
26	0.39279893	13	99.9959%								
27	0.453141	7	99.9977%								
28	0.51489234	6	99.9992%								
31	0.69046849	1	99.9995%								
34	0.82419533	2	100.0000%								
