# Supplementary material for: Children Comorbidity Score, a Simple Predictor for In-hospital Mortality: A Nationwide Inpatient Database Study in Japan
Source: JMA J. 2025 Apr 4;8(2):568–79. doi: 10.31662/jmaj.2024-0333 (PMC12095624; doi:10.31662/jmaj.2024-0333)
Supplement: Supplementary File 2 — Children Comorbidity Score Stata Program. [file 2433-3298-8-2-0568-s002.pdf]

Supplementary File 2: Children Comorbidity Score Stata Program

Actual Stata code to apply the Children Comorbidity Score to user data.

```
program define icd10_ccs
*! Version: 1.0.0
*! Author:   Nobuaki Michihata
*! Date:     January 26, 2024

version 11.0

syntax varlist (min=1)

di "Updating dataset..." _newline(2)

tempvar neuro cardio resp hemato congenital transplant malignant neonatal device gastro
score
gen `neuro'      = 0
gen `cardio'     = 0
gen `resp'       = 0
gen `hemato'     = 0
gen `congenital' = 0
gen `transplant' = 0
gen `malignant' = 0
gen `neonatal'   = 0
gen `device'     = 0
gen `gastro' = 0


qui foreach var of varlist `varlist' {
* Neurological and neuromuscular
replace `neuro' = 1 if inlist(substr(`var',1,3), "F71", "F72", "F73", "F79", "G10", "G11",
"G12", "G13", "G20")
replace `neuro' = 1 if inlist(substr(`var',1,3), "G21", "G22", "G32", "G35", "G36", "G37",
"G40", "G41", "G71")
replace `neuro' = 1 if inlist(substr(`var',1,3), "G72", "G80", "G81", "G82", "G94", "Q00",
"Q01", "Q02", "Q03")
replace `neuro' = 1 if inlist(substr(`var',1,3), "Q04", "Q05", "Q06", "Q07", "R56")
replace `neuro' = 1 if inlist(substr(`var',1,4), "E750", "E751", "E752", "E754", "F842")
replace `neuro' = 1 if inlist(substr(`var',1,4), "G041", "G230", "G231", "G232", "G238",
"G248")
replace `neuro' = 1 if inlist(substr(`var',1,4), "G253", "G254", "G255", "G259", "G312",
"G318", "G319", "G830", "G831")
replace `neuro' = 1 if inlist(substr(`var',1,4), "G832", "G833", "G834", "G835", "G839",
"G901", "G909", "G911", "G931")
replace `neuro' = 1 if inlist(substr(`var',1,4), "G934", "G935", "G938", "G939", "Q851",
```

```
"R403", "R470", "Z982")

* Cardiovascular
replace `cardio' = 1 if inlist(substr(`var',1,2), "I6")
replace `cardio' = 1 if inlist(substr(`var',1,3), "G45", "G46", "I05", "I06", "I07", "I08",
"I10", "I11", "I12")
replace `cardio' = 1 if inlist(substr(`var',1,3), "I13", "I15", "I21", "I22", "I26", "I27", "I34",
"I35", "I36")
replace `cardio' = 1 if inlist(substr(`var',1,3), "I37", "I38", "I39", "I42", "I43", "I44", "I45",
"I47", "I48")
replace `cardio' = 1 if inlist(substr(`var',1,3), "I49", "I50", "I70", "I71", "Q20", "Q22",
"Q23", "Q24", "Q26")
replace `cardio' = 1 if inlist(substr(`var',1,4), "A520", "H340", "I091", "I098", "I099",
"I252", "I255", "I280", "I288")
replace `cardio' = 1 if inlist(substr(`var',1,4), "I289", "I515", "I517", "I731", "I738",
"I739", "I771", "I790", "I792")
replace `cardio' = 1 if inlist(substr(`var',1,4), "K551", "K558", "K559", "P290", "Q212",
"Q213", "Q214", "Q218", "Q219")
replace `cardio' = 1 if inlist(substr(`var',1,4), "Q251", "Q252", "Q253", "Q254", "Q255",
"Q256", "Q257", "Q258", "Q259")
replace `cardio' = 1 if inlist(substr(`var',1,4), "Q282", "Q283", "Q289", "R000", "R001",
"R008", "T821", "Z450", "Z941")
replace `cardio' = 1 if inlist(substr(`var',1,4), "Z950", "Z951", "Z952", "Z953", "Z954",
"Z958", "Z959")

* Respiratory
replace `resp' = 1 if inlist(substr(`var',1,2), "J4")
replace `resp' = 1 if inlist(substr(`var',1,3), "E84", "I43", "J60", "J61", "J62", "J63", "J64",
"J65")
replace `resp' = 1 if inlist(substr(`var',1,3), "J66", "J67", "Q30", "Q31", "Q32", "Q33",
"Q34")
replace `resp' = 1 if inlist(substr(`var',1,4), "I278", "I279", "J684", "J701", "J703", "P280",
"Z430", "Z902", "Z930")
replace `resp' = 1 if inlist(substr(`var',1,4), "Z942", "Z990")

* Hematologic and immunologic
replace `hemato' = 1 if inlist(substr(`var',1,3), "B20", "B21", "B22", "B23", "B24", "D51",
"D52", "D53", "D55")
replace `hemato' = 1 if inlist(substr(`var',1,3), "D56", "D57", "D58", "D60", "D61",
"D64", "D65", "D66", "D67")
replace `hemato' = 1 if inlist(substr(`var',1,3), "D68", "D71", "D80", "D81", "D82",
"D83", "D84", "D85", "D86")
replace `hemato' = 1 if inlist(substr(`var',1,3), "D87", "D88", "M05", "M06", "M08",
"M30", "M32", "M33", "M34")
replace `hemato' = 1 if inlist(substr(`var',1,3), "M35", "M45", "Z21")
replace `hemato' = 1 if inlist(substr(`var',1,4), "D500", "D508", "D509", "D691", "D693",
"D694", "D695", "D696", "D700")
```

```
replace `hemato' = 1 if inlist(substr(`var',1,4), "D704", "D720", "D761", "D762", "D763", "L940", "L941", "L943", "M120")
replace `hemato' = 1 if inlist(substr(`var',1,4), "M123", "M310", "M311", "M312", "M313", "M314", "M315", "M316", "M360")
replace `hemato' = 1 if inlist(substr(`var',1,4), "M461", "M468", "M469")

* Other congenital or genetic defect
replace `congenital' = 1 if inlist(substr(`var',1,3), "Q77", "Q81", "Q87", "Q93", "Q97", "Q98")
replace `congenital' = 1 if inlist(substr(`var',1,4), "E343", "K449", "M410", "M412", "M418", "M419", "M965", "Q722", "Q750")
replace `congenital' = 1 if inlist(substr(`var',1,4), "Q752", "Q759", "Q760", "Q761", "Q762", "Q764", "Q765", "Q766", "Q767")
replace `congenital' = 1 if inlist(substr(`var',1,4), "Q780", "Q781", "Q782", "Q783", "Q784", "Q788", "Q789", "Q790", "Q791")
replace `congenital' = 1 if inlist(substr(`var',1,4), "Q792", "Q793", "Q794", "Q795", "Q799", "Q897", "Q898", "Q899", "Q909")
replace `congenital' = 1 if inlist(substr(`var',1,4), "Q913", "Q914", "Q917", "Q928", "Q950", "Q969", "Q992", "Q998", "Q999")

* Transplantation
replace `transplant' = 1 if inlist(substr(`var',1,3), "T86", "Z94")

* Malignant
replace `malignant' = 1 if inlist(substr(`var',1,1), "C")
replace `malignant' = 1 if inlist(substr(`var',1,3), "D00", "D01", "D02", "D03", "D04", "D05", "D06", "D07", "D08")
replace `malignant' = 1 if inlist(substr(`var',1,3), "D09", "D37", "D38", "D39", "D40", "D41", "D42", "D43", "D44")
replace `malignant' = 1 if inlist(substr(`var',1,3), "D45", "D46", "D47", "D48", "D49")
replace `malignant' = 1 if inlist(substr(`var',1,4), "Q850")

* Premature and neonatal
replace `neonatal' = 1 if inlist(substr(`var',1,3), "P84")
replace `neonatal' = 1 if inlist(substr(`var',1,4), "P052", "P059", "P100", "P101", "P104", "P115", "P210", "P219", "P250")
replace `neonatal' = 1 if inlist(substr(`var',1,4), "P251", "P253", "P258", "P270", "P271", "P278", "P350", "P351", "P524")
replace `neonatal' = 1 if inlist(substr(`var',1,4), "P528", "P560", "P570", "P578", "P613", "P614", "P773", "P832", "P912")
replace `neonatal' = 1 if inlist(substr(`var',1,4), "P916")

* Use of medical devices
replace `device' = 1 if inlist(substr(`var',1,4), "T865", "T872", "Y831", "Y833", "Z430", "Z431", "Z432", "Z433", "Z434")
replace `device' = 1 if inlist(substr(`var',1,4), "Z435", "Z436", "Z446", "Z930", "Z931", "Z932", "Z933", "Z934", "Z936")
```

```
replace `device' = 1 if inlist(substr(`var',1,4), "Z940", "Z942", "Z950", "Z952", "Z953", "Z959", "Z982", "Z990", "Z992")


* Gastrointestinal
replace `gastro' = 1 if inlist(substr(`var',1,3), "B18", "I85", "K21", "K25", "K26", "K27", "K28", "K50", "K51")
replace `gastro' = 1 if inlist(substr(`var',1,3), "K70", "K72", "K73", "K74", "Q41", "Q42", "Q43", "Q44", "Q45")
replace `gastro' = 1 if inlist(substr(`var',1,4), "I820", "I864", "I982", "K551", "K562", "K593", "K711", "K713", "K714")
replace `gastro' = 1 if inlist(substr(`var',1,4), "K715", "K717", "K754", "K760", "K761", "K762", "K763", "K764", "K765")
replace `gastro' = 1 if inlist(substr(`var',1,4), "K766", "K767", "K768", "K769", "Q390", "Q391", "Q392", "Q393", "Q394")
replace `gastro' = 1 if inlist(substr(`var',1,4), "Z431", "Z432", "Z433", "Z434", "Z903", "Z931", "Z932", "Z933", "Z934")
replace `gastro' = 1 if inlist(substr(`var',1,4), "Z944", "Z980")


gen `score' = `neuro' * 8 + `cardio' * 6 - `resp' * 3 + `hemato' * 7 + `congenital' * 3+ `transplant' * 6 + `malignant' * 10 + `neonatal' * 5 + `device' * 4 + `gastro' + 3

capture drop cs_score cs_neuro cs_cardio cs_resp cs_hemato cs_congenital cs_transplant cs_malignant cs_neonatal cs_device cs_gastro
gen cs_score      = `score'
gen cs_neuro      = `neuro'
gen cs_cardio     = `cardio'
gen cs_resp       = `resp'
gen cs_hemato     = `hemato'
gen cs_congenital = `congenital'
gen cs_transplant = `transplant'
gen cs_malignant  = `malignant'
gen cs_neonatal   = `neonatal'
gen cs_device     = `device'
gen cs_gastro     = `gastro'


di "Variables successfully added:"
di "- cs_score"
di "- cs_neuro"
di "- cs_cardio"
di "- cs_resp"
di "- cs_hemato"
di "- cs_congenital"
di "- cs_transplant"
di "- cs_malignant"
```

```
di "- cs_neonatal"
di "- cs_device"
di "- cs_gastro"
di ""
di "Dataset updated. New variables are now available for analysis."

end
```
