# Supplementary figures and images for: Children Comorbidity Score, a Simple Predictor for In-hospital Mortality: A Nationwide Inpatient Database Study in Japan
Source: JMA J. 2025 Apr 4;8(2):568–79. doi: 10.31662/jmaj.2024-0333 (PMC12095624; doi:10.31662/jmaj.2024-0333)

Supplementary  
Figure 1

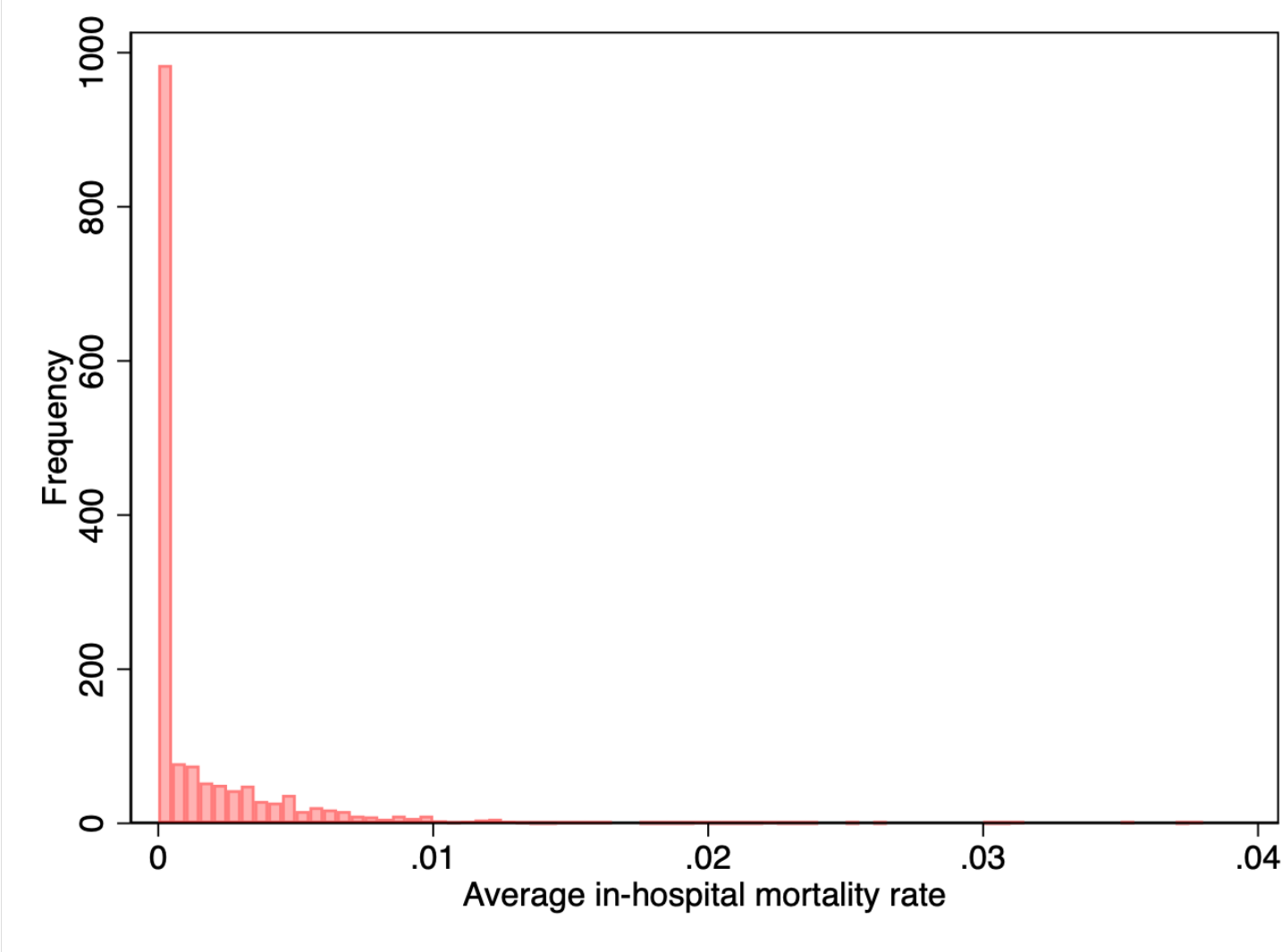

Supplementary  
Figure 2

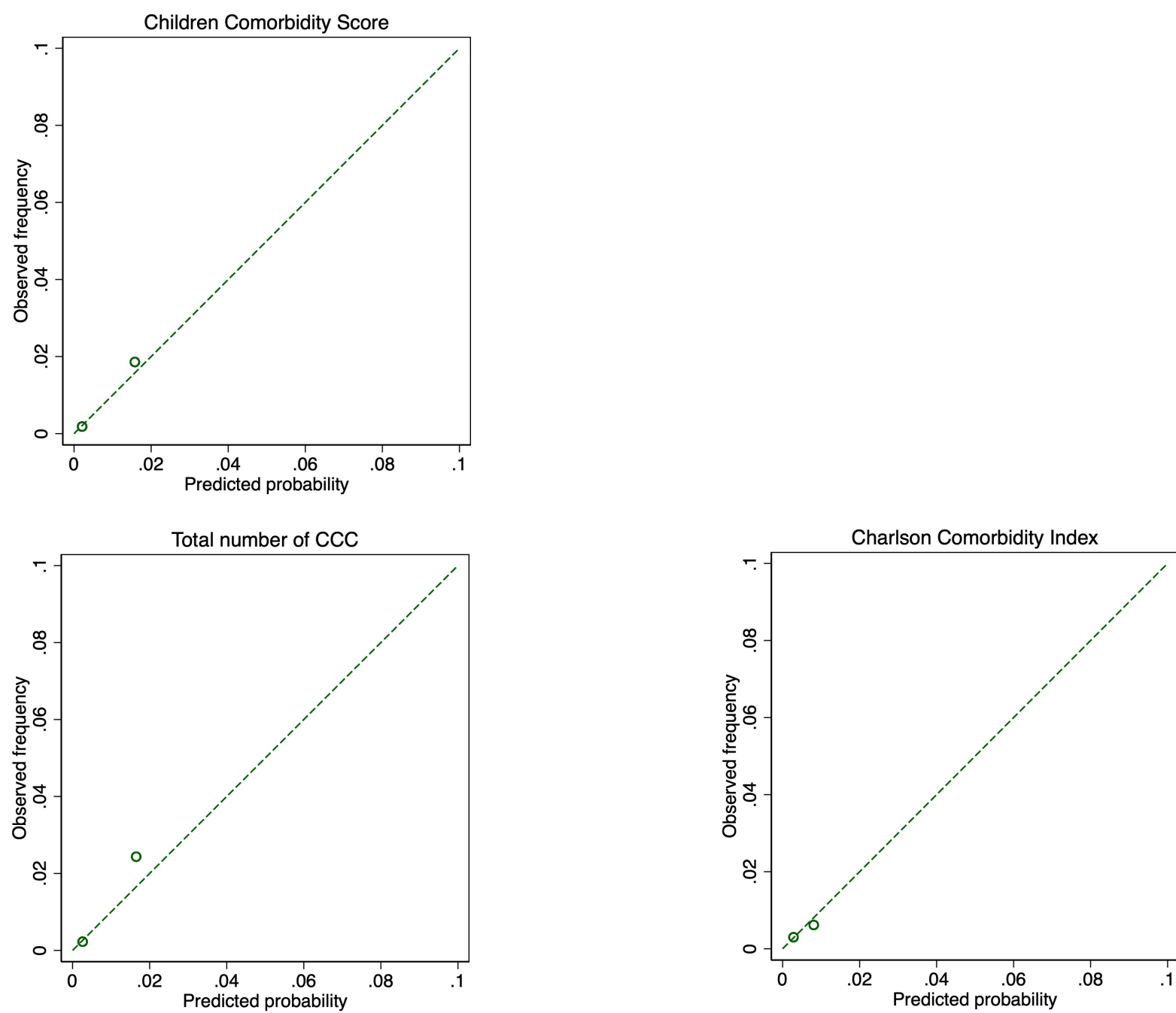

Supplementary  
Figure 3

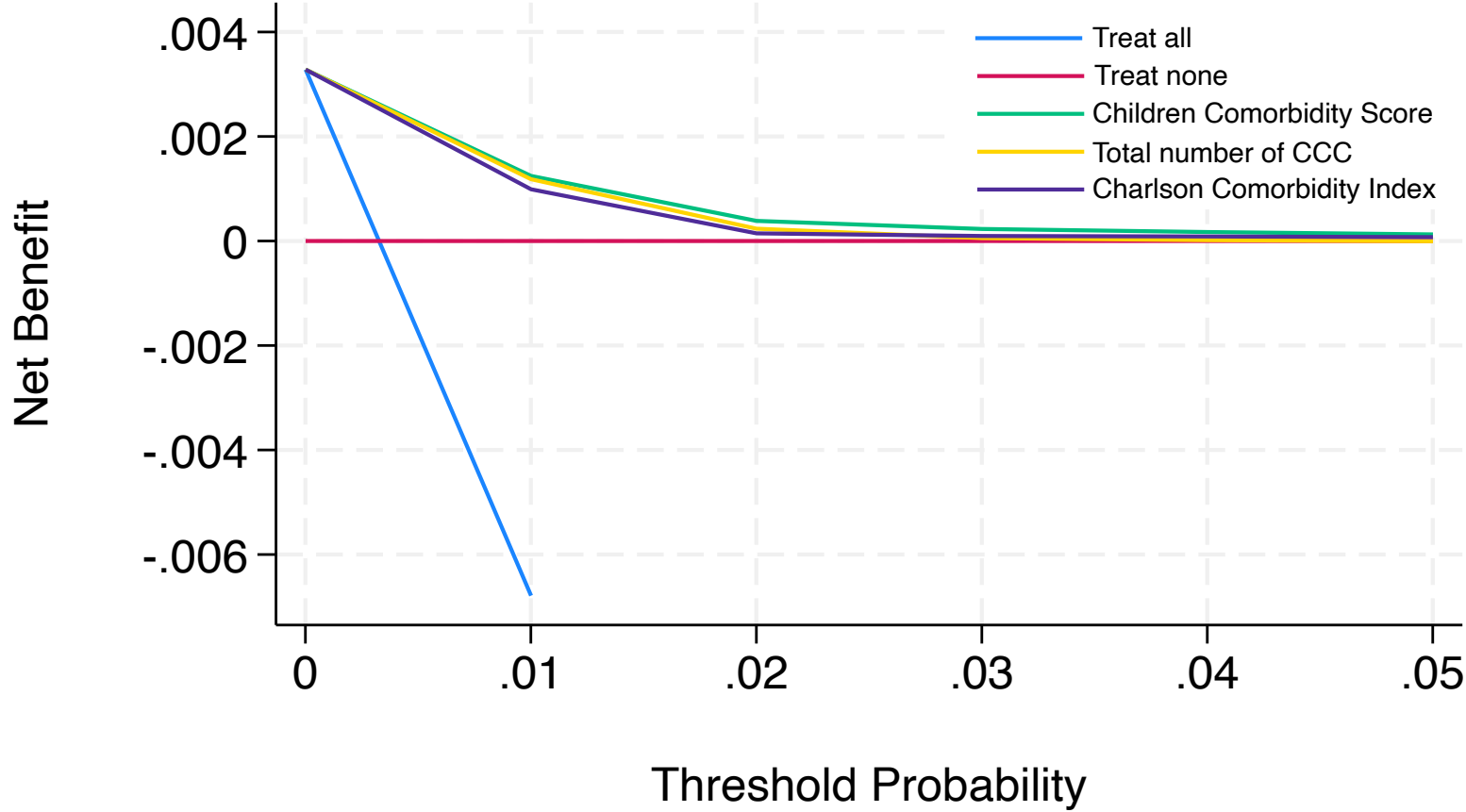

Supplement: Supplementary Figures — Supplementary Figure 1 Histogram of the average in-hospital mortality rate per hospital. Histogram of the average in-hospital mortality rate per hospital. For clarity, this figure includes data up to the 99th percentile of the total number of hospitals. Supplementary Figure 2 Calibration plots of the Children Comorbidity Score and reference models for predicting in-hospital mortality. Due to the low probability of in-hospital mortality, we presented a plot limited to the display range of predicted probabilities up to 0.1. Supplementary Figure 3 Decision curve analysis of the developed model (Children Comorbidity Score) and reference models for predicting in-hospital mortality including two extreme default scenarios. The “treat none” line represents a scenario where no patients receive preventive care for the risk of in-hospital death, avoiding unnecessary interventions but failing to support any high-risk patients, resulting in a net benefit of zero across all risk thresholds. On the other hand, the “treat all” line represents a scenario where all patients are prepared for the risk of in-hospital death, regardless of their risk, ensuring no high-risk cases are missed but leading to unnecessary interventions for low-risk patients. [file 2433-3298-8-2-0568-s003.pdf]
